# Acute and Chronic Toxicity Tests and Assessment of In Vitro and In Vivo Anti-Allergic Effects of Prabchompoothaweep Remedy

**DOI:** 10.3390/ijms27157031

**Published:** 2026-08-05

**Authors:** Sunita Makchuchit, Pattarapol Pusiripinyo, Areeratna Jai-uea, Pranporn Kuropakornpong, Seewaboon Sireeratawong, Parirat Khonsung, Neal M. Davies, Arunporn Itharat

**Affiliations:** 1 Thammasat Postdoctoral Fellowship, Thammasat University, Klongluang, Pathumthani 12120, Thailand; msunita_359@hotmail.com; 2 Center of Excellence in Applied Thai Traditional Medicine Research (CEATMR), Thammasat University, Klongluang, Pathumthani 12120, Thailand; pranporn.kur@gmail.com; 3 Department of Applied Thai Traditional Medicine, Faculty of Medicine, Thammasat University, Klongluang, Pathumthani 12120, Thailand; pattarapol.attm@gmail.com (P.P.); areeratanajai@gmail.com (A.J.-u.); 4 Department of Pharmacology, Faculty of Medicine, Chiang Mai University, Chiang Mai 50200, Thailand; seewaboon@gmail.com (S.S.); parirat.khons@cmu.ac.th (P.K.); 5 Clinical Research Center for Food and Herbal Product Trials and Development (CR-FAH), Faculty of Medicine, Chiang Mai University, Chiang Mai 50200, Thailand; 6 Faculty of Pharmacy and Pharmaceutical Sciences, Katz Group-Rexall Centre for Pharmacy and Health Research, University of Alberta, Edmonton, AB T6G 2E1, Canada; ndavies@ualberta.ca

**Keywords:** toxicity, allergic rhinitis, Prabchompoothaweep, β-hexosaminidase, ovalbumin, Thai traditional medicine

## Abstract

Prabchompoothaweep (PCT) is an anti-allergic remedy on the Thailand National List of Essential Medicines, traditionally used to relieve common colds and allergic reactions in Thai traditional medicine (TTM). However, the anti-allergic activity of PCT and its plant ingredients had not been characterized. We evaluated the acute and chronic oral toxicity of PCT ethanolic extract in rats and assessed its in vitro and in vivo anti-allergic activities. Activity in vitro was measured as inhibition of antigen-induced β-hexosaminidase release in RBL-2H3 cells and in vivo using an ovalbumin (OVA)-induced allergic rhinitis mouse model. The extract produced no mortality or signs of toxicity in either study. Several plant ingredients and the PCT extract inhibited β-hexosaminidase release more effectively than chlorpheniramine. In OVA-induced mice, PCT (75, 150, and 300 mg/kg) did not significantly lower OVA-specific serum IgE or IgG2a (OVA-specific IgG1 was likewise not reduced); however, it reduced inflammatory cell infiltration, goblet cell hyperplasia and mast cell numbers in the nasal mucosa and downregulated the T helper 2 (Th2) cytokines IL-5 and IL-13. These findings support the traditional anti-allergic use of the PCT remedy.

## 1. Introduction

Allergic rhinitis (AR) is a common chronic disease characterized by symptoms such as nasal itching, sneezing, rhinorrhea, and nasal congestion [1]. It affects up to 40% of the world’s population, causing significant morbidity and impairing quality of life [2]. AR is an IgE-mediated response against inhaled allergens that induces inflammation via Th2 cells. The allergic response occurs in two phases, early and late, with histamine being one of the primary mediators of allergic rhinitis [2,3,4,5]. Various therapeutic options are available for AR treatment, but they often have adverse side effects [6,7]. Medicinal plants have regained popularity as alternative and complementary treatments for AR due to their safety and reduced side effect profile [8].

Prabchompoothaweep (PCT) is a popular traditional remedy on the Thailand National List of Essential Medicines (NLEM) used to relieve symptoms related to respiratory tract infections and allergies [9]. This remedy consists of twenty-three medicinal plants, including *Acanthus ebracteatus* Vahl. (Ngueak pla mo), *Amomum testaceum* Ridl. (Krawan), *Amorphophallus saraburiensis* (Dennst.) (Buk ror), *Anethum graveolens* L. (Thian ta takkataen), *Angelica dahurica* Benth. (Kot sor), *Ardisia polycephala* Wall. ex A.DC. (Philangkasa), *Atractylodes lancea* (Thunb.) DC. (Kot kamao), *Cinnamomum camphora* (L.) J.S. Presl (Karabun), *Clausena excavata* Burm. f. (Hasakhun thet), *Enhalus acoroides* (L.f) Rovle. (Lamphan hangmu), *Foeniculum vulgare* Mill. (Thian klaep), *Leonurus sibiricus* L. (Kancha thet), *Lepidium sativum* L. (Thian daeng), *Myristica fragrans* Houtt. (Look chan or Nutmeg), *Myristica fragrans* Houtt. (Dok chan or Mace), *Nigella sativa* L. (Thian dam), *Piper retrofractum* Vahl. (Dee plee), *Piper nigrum* L. (Phrikthai dam), *Plumbago indica* L. (Chetta mun phloeng daeng), *Syzygium aromaticum* (L.) Merr. & L.M. Perry (Kan phlu), *Terminalia arjuna* Wight and Arn. (Samo thet), *Terminalia chebula* Retz. (Samo thai) and *Zingiber officinale* Roscoe (Khing). The PCT remedy is a polyherbal remedy that stimulates the cardiovascular and lymphatic systems, reduces inflammation of the skin and respiratory tract, and uses the hot and spicy flavor of its ingredients to enhance its allergy treatment. In recent years, the PCT remedy has not only been reported to have in vitro antioxidant [10], anti-inflammatory [10], anti-allergic [10], and antimalarial activities [11]; it has also exhibited in vivo anti-inflammatory [12] and antimalarial activities [13]. However, there is very little information available about the toxicity of the PCT, and neither the in vitro anti-allergic activity of plant ingredients of the PCT remedy nor the in vivo anti-allergic activity of the PCT remedy have yet been investigated.

Therefore, this study aimed to evaluate the acute and chronic toxicities in rats. The experiments examined not only the effects of plant ingredients in the PCT remedy on antigen-induced IgE-sensitized degranulation of rat basophilic leukemia cells but also the allergic activities of the PCT remedy in an ovalbumin-induced allergic rhinitis mouse model. We evaluated the therapeutic effect of the PCT in reducing inflammatory parameters, including OVA-specific IgE, IgG1, and IgG2a levels, infiltration of inflammatory cells, goblet cell hyperplasia, mast cell infiltration, and cytokine expression in nasal mucosa. These findings provide information on the safety of the PCT to provide guidance for selecting a safe dose for clinical application and suggest that PCT may have therapeutic potential for the treatment of allergy-related diseases, such as allergic rhinitis.

## 2. Results

### 2.1. Extraction Yields of PCT Remedy and Its Plant Ingredients

The proportions of each plant used in the Prabchompoothaweep remedy are shown in Table S1. The yield of the ethanolic extract of the PCT remedy was 12.80%. It was dark brown. Additionally, the yields of all plant ingredient extracts were in the range of 1.44–46.38%. The extract with the highest yield was from *T*. *chebula*, which was 46.38%. All data on the yield are shown in Table S2. To support batch-to-batch reproducibility, an HPLC chemical fingerprint of the ethanolic PCT extract, together with quantification of the piperine marker compound (e.g., piperine from *Piper nigrum*, the main component in PCT remedy), is shown in Figure S1. The chemical fingerprint of PCT as determined by HPLC showed that the main ingredient of the PCT remedy was piperine, and its content was 50.85 ± 0.35 µg/g extract.

### 2.2. Acute Toxicity

The results of the preliminary toxicity study of the PCT remedy according to the Hippocratic screening test were obtained by orally giving an extract of 2000 mg/kg to female rats (*n* = 2). Then, changes in the general behavior and symptoms of rats within the first 24 h after receiving the extract were observed. It was found that the female rats did not behave abnormally compared to the control group that received distilled water, and no rat deaths were observed. Moreover, in the necropsies, no abnormalities were found in the color and size of the internal organs.

The results of the acute toxicity study of PCT according to the OECD guideline were obtained by giving a single oral dose of the PCT (5000 mg/kg) to female rats once (*n* = 5). After 24 h, it was found that none of the rats showed abnormal behavior compared with the control group. In addition, it was found that food consumption, body weight, and excretion in all rats did not differ from the control group. An oral administration of PCT (5000 mg/kg) showed no mortality. During the 14 days of observation, the single oral dose of PCT remedy (5000 mg/kg) did not cause mortality in any rats. No changes in general behavior were observed. The body weight on days 7 and 14 of the rats in the PCT-treated group had significantly decreased compared with the control group (*p* < 0.05) (Table S3). As shown in Table S4, the reduction in lung, heart, liver, spleen, kidney, and uterus weights of rats in the PCT-treated group showed a significant difference from the control group (*p* < 0.05). However, in the necropsies, the color and size of the internal organs were normal.

### 2.3. Chronic Toxicity

**Figure 1:**
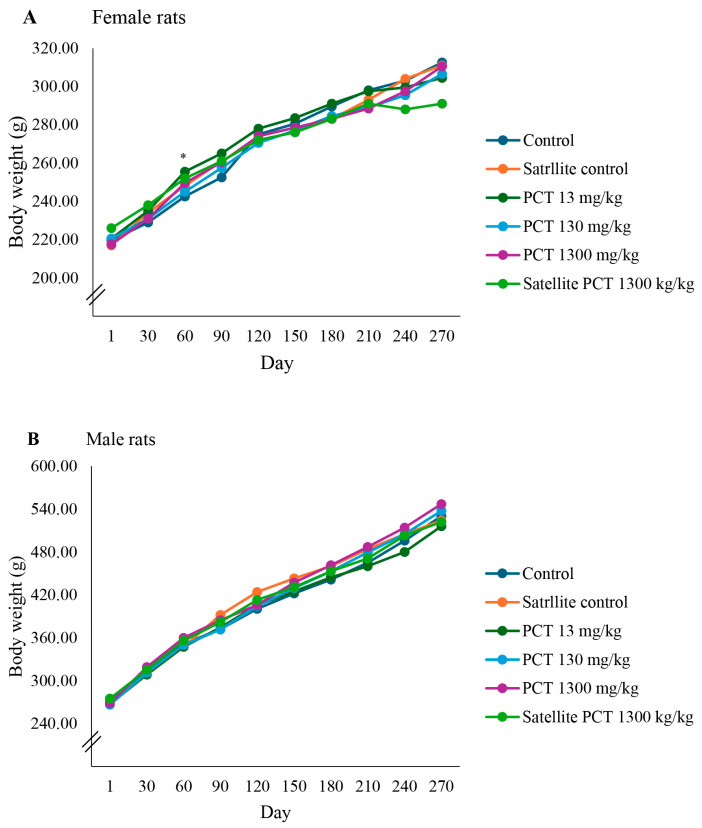
Body weight of female rats (**A**) and male rats (**B**) treated with PCT in chronic toxicity. * Significantly different from control (*p* < 0.05).

The results of the chronic toxicity study in which male and female rats were fed PCT (13, 130, and 1300 mg/kg) every day for 270 days and observed for changes in general behavior and symptoms are presented below. On day 270, it was found that there were no abnormal behaviors compared to the control group. No changes in food intake, body weight, and excretion in female and male rats were observed compared to the control group, and no mortality was found in any group. The body weight of female and male rats receiving all doses of PCT is presented in Figure 1. Only female rats receiving PCT (13 mg/kg) showed a significant increase in body weight at day 60 compared to the control group (*p* < 0.05). Meanwhile, the body weight of male rats receiving all doses of PCT did not differ significantly from the control group. The organ weight of female and male rats is revealed in Table 1. Female rats who received all doses of PCT (13, 130, and 1300 mg/kg) showed no significant difference in organ weight compared to the control group, while the satellite groups of the control group had a significant increase in heart and liver weight compared to the control group (*p* < 0.05). Likewise, the satellite groups of the PCT (1300 mg/kg)-treated group had significantly decreased brain and kidney weight and significantly increased heart and liver weight compared to the control group (*p* < 0.05). In male rats fed 13 mg/kg of PCT, testis weight was significantly lower than that of the control group (*p* < 0.05). Additionally, the satellite groups of the control group had significantly decreased lung and liver weights compared to the control group (*p* < 0.05), while the satellite groups of the PCT-treated group had significantly decreased lung, liver, and testis weights (*p* < 0.05).

The results from the hematological parameters in the female and male rats are depicted in Table 2. In female rats, platelet counts (PLTs) in the PCT (130 mg/kg) group were significantly lower than those in the control group (*p* < 0.05). The HCT and MCV values in the PCT (1300 mg/kg) group were significantly increased compared with the control group (*p* < 0.05). Furthermore, the levels of RBC, HB, and HCT in the PCT satellite group (1300 mg/kg) were significantly increased. Moreover, the PCT (13 mg/kg) group had significantly reduced MONO counts, while the PCT (130 mg/kg) group had significantly reduced LYMP and MONO counts compared with the control group (*p* < 0.05). In addition, the PCT (1300 mg/kg) group had significantly increased amounts of NEU compared with the control group (*p* < 0.05). In male rats treated with PCT (130 mg/kg), only the level of HCT was significantly increased compared to the control group (*p* < 0.05). Further, the amounts of WBC and EOS were significantly decreased in the PCT satellite group (1300 mg/kg).

The blood biochemistry values of the female and male rats are shown in Table 3. In female rats given all doses of PCT (13, 130, and 1300 mg/kg), GLU, BUN, CRE, TP, ALB, T-BIL, D-BIL, AST, ALT, and ALP levels showed no differences from the control group, but the level of TP in the PCT satellite group (1300 mg/kg) was significantly increased, and the level of ALP in the PCT group at the 130 mg/kg dose was significantly decreased. In male rats, the level of GLU was significantly reduced in the PCT (13 and 130 mg/kg) and the satellite PCT (1300 mg/kg) groups. In addition, the levels of T-BIL and D-BIL were significantly increased in both the satellite group of the control group and the satellite group of the PCT (1300 mg/kg) group compared with the control group (*p* < 0.05). Because a large number of hematological and biochemical parameters were compared across the treatment groups, the isolated, non-dose-related differences that reached statistical significance, all of which remained within the reference ranges for the strain, should be interpreted with caution and are unlikely to represent treatment-related effects.

**Figure 2:**
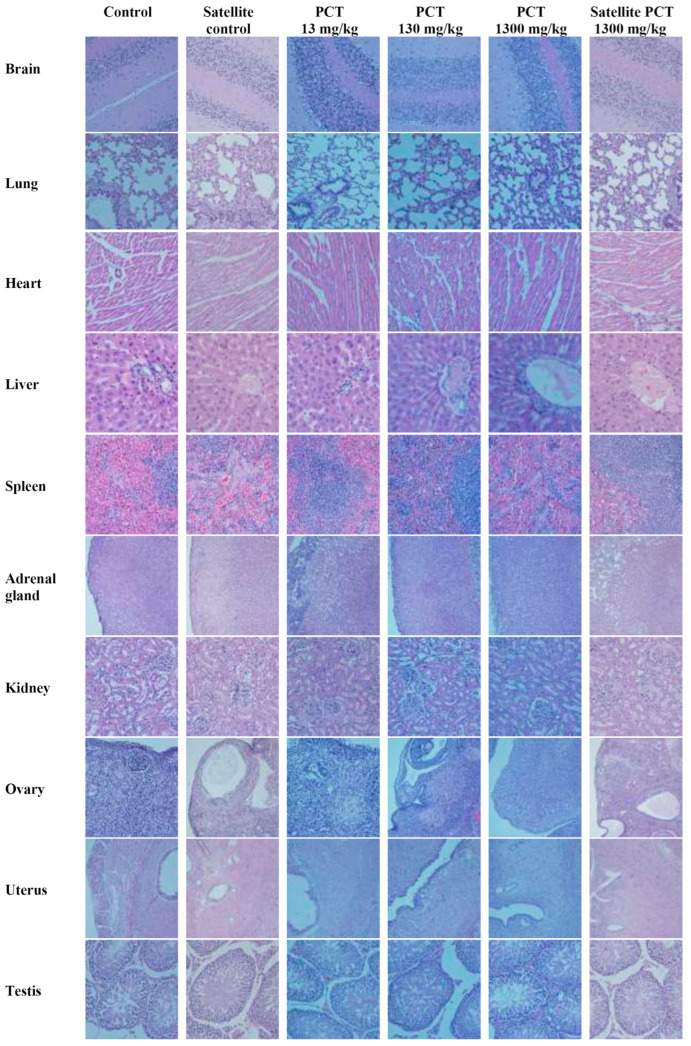
Histological sections of brain, lung, heart, liver, spleen, adrenal gland, kidney, ovary, uterus, and testis of rats in chronic toxicity (40× magnification, hematoxylin and eosin staining).

No significant pathologic changes were observed in the tissue sections of the brain, lungs, heart, liver, spleen, adrenal glands, kidneys, and sex organs of rats treated with PCT at doses of 13, 130, and 1300 mg/kg (Figure 2).

### 2.4. In Vitro Anti-Allergic Activity on β-Hexosaminidase Release in IgE-Stimulated RBL-2H3 Cells

**Figure 3:**
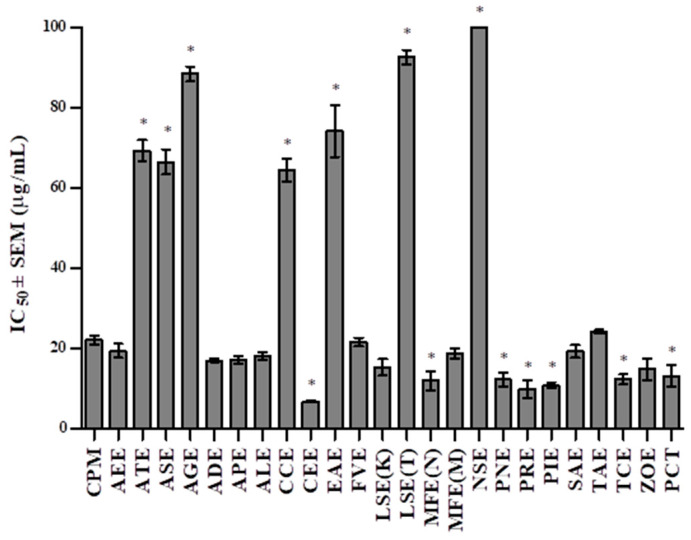
Inhibitory effects of the PCT ethanolic extract, its plant ingredients, and the positive control drug (Chlorpheniramine, CPM) on the IgE-stimulated degranulation response of RBL-2H3 cells. The data were analyzed by one-way ANOVA and Dunnett’s multiple comparison tests. * *p* < 0.05 compared with CPM.

All extracts were also tested for inhibitory effects on the IgE-stimulated degranulation response of RBL-2H3 cells in comparison with chlorpheniramine (CPM). The results are shown in Table S2 and Figure 3. PCT remedy extract inhibited β-hexosaminidase release, with an IC_50_ value of 13.18 ± 2.67 µg/mL. This IC_50_ value was significantly lower (i.e., more potent) than that of the positive control drug, CPM (IC_50_ value of 22.12 ± 1.07 µg/mL) (*p* < 0.05). The ethanolic extracts of *C*. *excavata* (CEE), *P*. *retrofractum* (PRE), *P*. *indica* (PIE), *M*. *fragrans* (Nutmeg) (MFE(N)), *P*. *nigrum* (PNE), and *T*. *chebula* (TCE) presented a potent inhibition effect on the release of β-hexosaminidase with IC_50_ values of 6.82 ± 0.23, 9.97 ± 2.15, 10.88 ± 0.66, 12.04 ± 2.41, 12.30 ± 1.85, and 12.37 ± 1.25 µg/mL, respectively. They were significantly different from CPM (*p* < 0.05). Additionally, the results found that *Z*. *officinale* (ZOE), *L*. *sibiricus* (LSE(K)), *A*. *dahurica* (ADE), *A*. *polycephala* (APE), *A*. *lancea* (ALE), *M*. *fragrans* (Mace) (MFE(M)), *S*. *aromaticum* (SAE), *A*. *ebracteatus* (AEE), *F*. *vulgare* (FVE), and *T*. *arjuna* (TAE) showed high anti-allergic activity on the inhibition of β-hexosaminidase release, with IC_50_ values lower than CPM, but they were not significantly different from the positive control drug (*p* > 0.05) (IC_50_ values of 14.84 ± 2.79, 15.35 ± 2.01, 17.08 ± 0.34, 17.17 ± 0.97, 18.15 ± 0.83, 18.86 ± 1.32, 19.31 ± 1.57, 19.49 ± 1.76, 21.68 ± 1.06, and 24.26 ± 0.55 µg/mL, respectively), except *T*. *arjuna* (TAE), which showed a higher IC_50_ value than CPM. On the other hand, the ethanolic extracts of *C*. *camphora* (CCE), *A*. *saraburiensis* (ASE), *A*. *testaceum* (ATE), *E*. *acoroides* (EAE), *A*. *graveolens* (AGE), and *L*. *sativum* (LSE(T)) possessed mild activity (IC_50_ ranged between 60 and 100 µg/mL).

### 2.5. In Vivo Anti-Allergic Activity of PCT Remedy

#### 2.5.1. Serum OVA-Specific IgE, IgG1, and IgG2a

**Figure 4:**
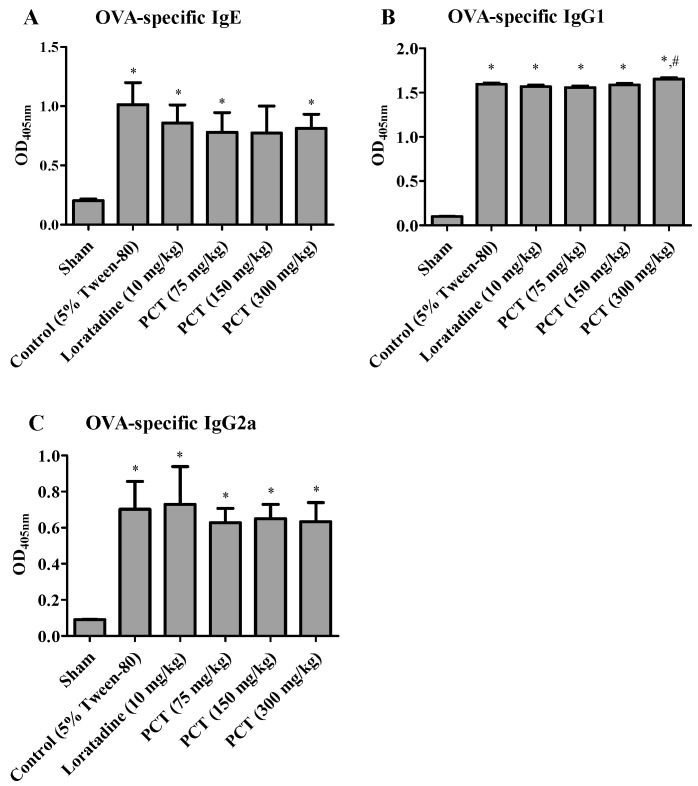
Effect of Prabchompoothaweep (PCT) and loratadine on the levels of (**A**) OVA-specific serum IgE; (**B**) OVA-specific serum IgG1; (**C**) OVA-specific serum IgG2a in serum of OVA-induced AR mice. The values are presented as the mean ± standard error of the mean (SEM) (*n* = 6 per group). The data were analyzed by one-way ANOVA and Dunnett’s multiple comparison tests. * *p* < 0.05 compared with the sham group; ^#^ *p* < 0.05 compared with the control group (allergenic mice).

Figure 4 shows the indirect ELISA OD_405nm_ values of OVA-specific IgE, IgG1, and IgG2a in the serum of OVA-induced AR mice compared to sham mice. The control group (sensitized mice with OVA) exhibited significantly higher levels of serum IgE, IgG1, and IgG2a than the sham group (1.015 ± 0.184, 1.595 ± 0.016, and 0.703 ± 0.153, respectively, vs. 0.203 ± 0.014, 0.100 ± 0.003, and 0.090 ± 0.001, respectively; *p* < 0.05). These results indicate that OVA potently induces allergic responses in mice through the OVA-specific IgE in serum.

In the loratadine (10 mg/kg) and PCT (75, 150, and 300 mg/kg)-treated mouse groups, the means ± SEM of the indirect ELISA OD_405nm_ values of OVA-specific IgE in serum did not significantly decrease compared to the control group (Figure 4A) (*p* > 0.05). For the means ± SEM of the indirect ELISA OD_405nm_ values of OVA-specific IgG1, only the PCT (300 mg/kg)-treated mice group had a significant increase in the levels of OVA-specific IgG1 in the serum compared to the control group (Figure 4B) (*p* < 0.05). The means ± SEM of the indirect ELISA OD_405nm_ values of OVA-specific IgG2a in the loratadine (10 mg/kg) and PCT (75, 150, and 300 mg/kg)-treated mouse groups did not significantly differ from the control group (Figure 4C) (*p* > 0.05).

#### 2.5.2. Histopathology of Nasal Mucosa of OVA-Induced AR Mice

**Figure 5:**
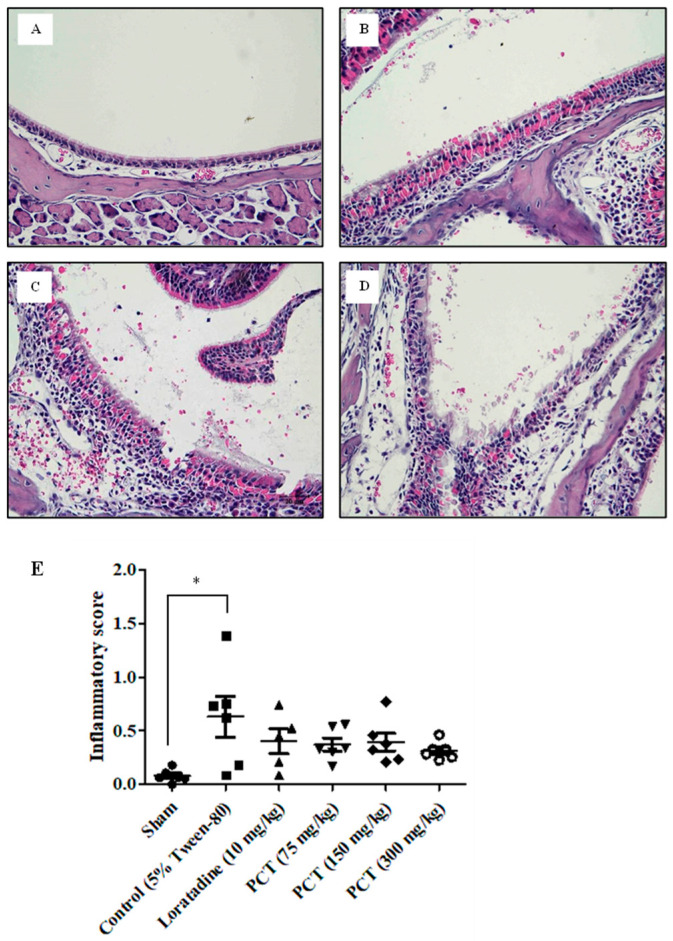
Histology and grades of nasal tissue sections of mice after staining with H&E dyes. (**A**–**D**) Histological grades 1–4, respectively. (**E**) Comparative average histological grades of sham, control, loratadine and PCT mice. The data were analyzed by one-way ANOVA and Dunnett’s multiple comparison tests. * *p* < 0.05 was considered significant as compared to the sham group.

**Figure 6:**
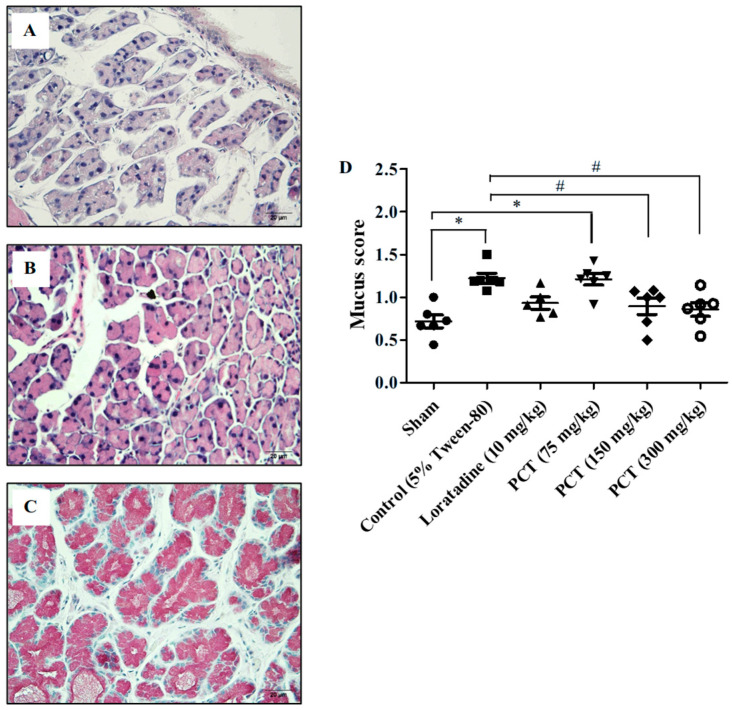
Histology and grades of nasal tissue sections of mice after staining with PAS dyes. (**A**–**C**) Histological grades 1–3 of color intensity of the PAS-stained mucus glands in the nasal tissues of mice, respectively. (**D**) Comparative average histological grades of sham, control, loratadine and PCT mice. The data were analyzed by one-way ANOVA and Dunnett’s multiple comparison tests. * *p* < 0.05 was considered significant as compared to the sham group. ^#^ *p* < 0.05 was considered significant as compared to the control group.

**Figure 7:**
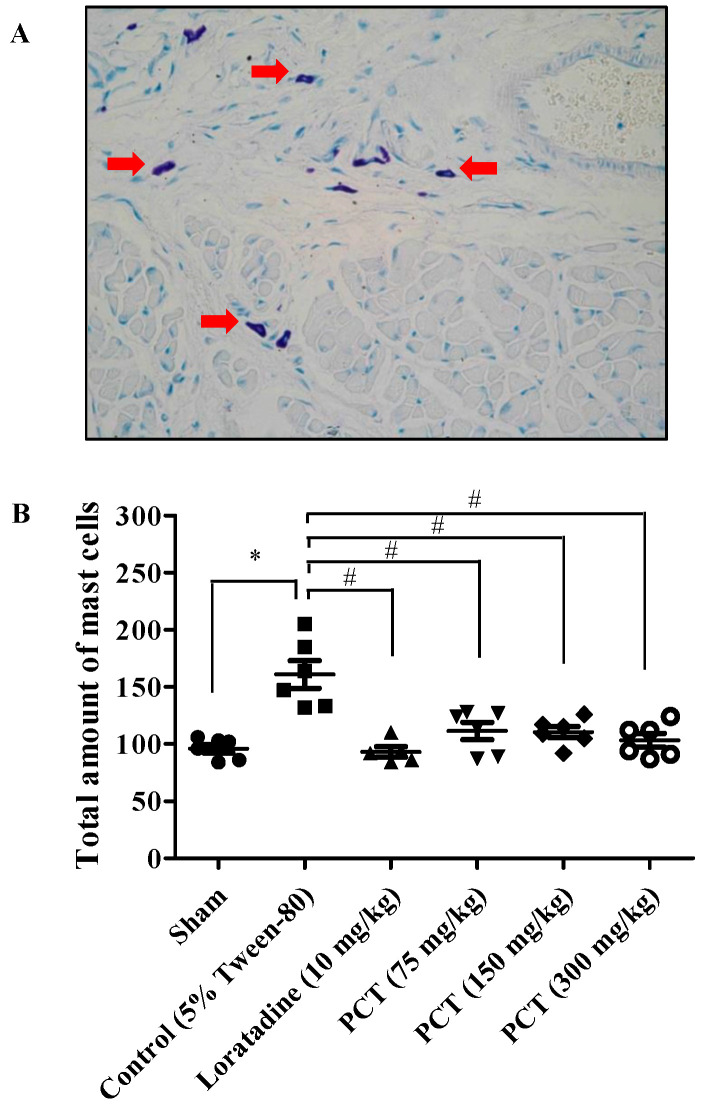
Histological appearances and total amount of mast cells of mice after staining with toluidine blue dye. (**A**) The mast cells (red arrows) in nasal tissue sections of mice. (**B**) Comparative average total amount of mast cells of sham, control, loratadine and PCT mice. The data were analyzed by one-way ANOVA and Dunnett’s multiple comparison tests. * *p* < 0.05 was considered significant as compared to the sham group. ^#^ *p* < 0.05 was considered significant as compared to the control group.

The infiltration of inflammatory cells into the mouse nasal mucosa was assessed by staining with H&E dyes, and the different grades (1–4) of inflammatory cells are shown in Table S5(A) and Figure 5. The results revealed that the average grade of nasal mucosa inflammation score in OVA-sensitized mice (control group) was significantly higher (0.631 ± 0.191) than in the sham group (0.077 ± 0.025) (*p* < 0.05). However, treatment with loratadine (10 mg/kg) and PCT (75, 150, and 300 mg/kg) reduced the average grades of infiltrated inflammatory cells into nasal mucosa tissue compared to the control group (average grade scores 0.404 ± 0.117, 0.371 ± 0.061, 0.395 ± 0.084, and 0.313 ± 0.034, respectively). However, there were no significant differences observed among the treatment groups (*p* > 0.05) (Figure 5E). To assess mucus production in the mouse nasal mucosa, sections were stained with PAS dye, and different color intensities (grades 1–3) of mucus glands are shown in Table S5(B) and Figure 6. The results indicated that the average grade of mucus production in the nasal tissue of OVA-exposed mice was significantly higher (1.224 ± 0.058) than in the sham mice (0.718 ± 0.074) (*p* < 0.05). However, treatment with PCT (150 and 300 mg/kg) significantly decreased the mucus gland intensity compared to the control group (*p* < 0.05) (average grade score 0.894 ± 0.096 and 0.858 ± 0.081, respectively). To enumerate mast cells, mouse nasal mucosa sections were stained with TB dye, and the number of mast cells infiltrated in nasal tissues is shown in Figure 7. The average number of mast cells in the nasal tissues of OVA-induced AR mice (control mice) was significantly higher (161.000 ± 12.031) than that of sham mice (96.167 ± 3.781) (*p* < 0.05). All treatment groups, including loratadine (10 mg/kg)- and PCT (75, 150, and 300 mg/kg)-treated allergic mice, showed a significantly decreased average number of mast cells compared with the control group (*p* < 0.05) (average number 93.200 ± 4.587, 111.500 ± 7.706, 110.667 ± 4.752, and 103.333 ± 6.009, respectively).

#### 2.5.3. Cytokine Gene Expression in the Nasal Mucosa Tissues

**Figure 8:**
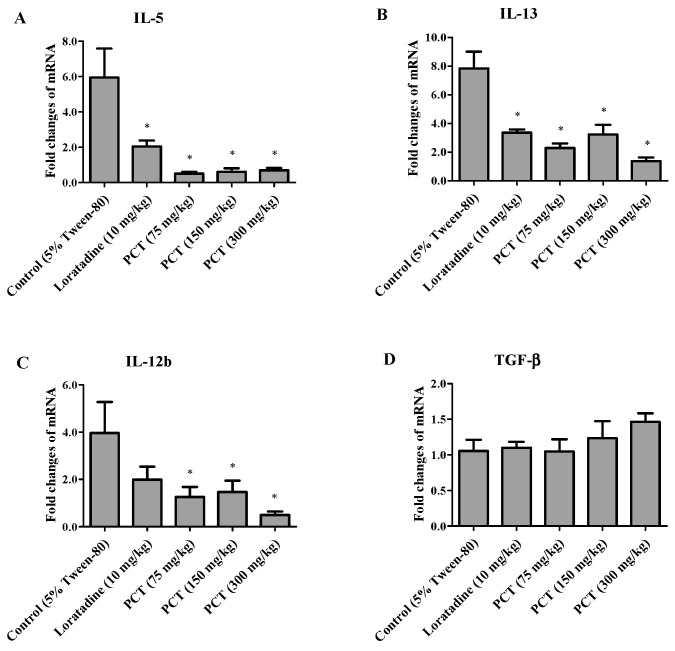
Fold changes in cytokine gene expression in nasal tissues of OVA-induced AR mice treated with loratadine and Prabchompoothaweep (PCT). The mRNA synthesis of (**A**) IL-5; (**B**) IL-13; (**C**) IL-12b; and (**D**) TGF-β in the nasal tissues of OVA-induced AR mice. Values are presented as the mean ± SEM (*n* = 6 per group). The data were analyzed by one-way ANOVA and Dunnett’s multiple comparison tests. * Significant difference (*p* < 0.05) when compared with the sham group.

Figure 8 displays the fold changes in mRNA expression of nasal tissues in allergic mice treated with different doses of loratadine (10 mg/kg) and PCT (75, 150, and 300 mg/kg) compared to the control group. The expression levels of IL-5 in the nasal tissues of allergic mice treated with loratadine (10 mg/kg) and PCT (75, 150, and 300 mg/kg) were significantly lower than those of the control group (5% Tween-80) (*p* < 0.05) (IL-5 levels 2.053 ± 0.339, 0.512 ± 0.089, 0.608 ± 0.211, 0.695 ± 0.132, and 5.952 ± 1.634, respectively) (Figure 8A). Moreover, the IL-5 expression levels of all PCT groups were lower than those of the loratadine-treated group, although the difference was not significant (*p* > 0.05). Similarly, IL-13 mRNA levels were significantly reduced in the nasal tissues of the loratadine (10 mg/kg) and PCT (75, 150, and 300 mg/kg) treatment groups (*p* < 0.05) (IL-13 levels 3.361 ± 0.230, 2.288 ± 0.312, 3.226 ± 0.687, 1.368 ± 0.258, and 7.835 ± 1.175, respectively) (Figure 8B). IL-12b mRNA levels in allergic mice treated with all PCT doses (75, 150, and 300 mg/kg) were significantly lower than those of the control group (*p* < 0.05) (Figure 8C), but not significantly lower than those of the loratadine group (*p* > 0.05) (IL-12b levels 1.256 ± 0.426, 1.468 ± 0.480, 0.490 ± 0.155, 3.966 ± 1.311, and 1.987 ± 0.557, respectively). There was no significant increase in TGF-β mRNA levels in the allergic mice treated with PCT (75, 150, and 300 mg/kg) (*p* > 0.05) (Figure 8D) compared to the control group (5% Tween-80) and the loratadine group (10 mg/kg) (TGF-β levels 1.048 ± 0.170, 1.235 ± 0.239, 1.463 ± 0.119, 1.054 ± 0.159, and 1.099 ± 0.085, respectively). These findings indicate that allergic mice treated with PCT (75, 150, and 300 mg/kg) had significantly reduced expression of Th2 cytokine genes (which are associated with allergic reactions) without significantly altering expression of the Treg cytokine gene TGF-β. Treg cytokines are associated with a decrease in allergic reactions upon allergen re-exposure. However, allergic mice treated with all PCT doses (75, 150, and 300 mg/kg) did not exhibit any stimulation or increase in Th1 cytokines.

## 3. Discussion

Toxicity testing is an evaluation of the safety profile of herbal products before their use in humans. The experiment aims to ascertain the toxicant dosages or concentrations, as well as the duration of exposure necessary to produce a specific effect. The criterion of effect may be death of the exposed organisms or a change in any other parameter, such as behavior, physiology, hematological, immunological, or biochemical parameters.

In acute toxicity testing, the test substance is administered in a single large oral dose or in multiple doses, and any adverse effects appearing within the first 24 h or 14 days of treatment are observed [14]. In this study, PCT remedy extract at a dose of 5000 mg/kg was selected for acute toxicity testing because an initial dose of 2000 mg/kg was preliminary tested and did not cause mortality or any signs or symptoms of toxicity. The results showed that a high dose of PCT did not result in abnormal symptoms, severe organ toxicity, or death. Although a significant difference in body weight loss in the PCT-treated group compared with the control group was observed. The reduction of body weight in the PCT-treated group reached statistical significance; although it was not accompanied by clinical signs of toxicity or by gross or histopathological organ abnormality, we treat it as a genuine treatment-related effect rather than dismissing it, and note that it may also be influenced by differences in food and water intake. Furthermore, a significantly lower weight of internal organs, including the lung, heart, liver, spleen, kidney, and uterus, was observed in the PCT-treated rats compared with the control rats, but neither gross nor histopathological abnormalities were examined in these organs. A previous report found that a 2000 mg/kg dose of PCT extract was not acutely toxic in ICR mice [12]. This is consistent with our study; we found that PCT was not acutely toxic. However, a higher dose of PCT (5000 mg/kg) and different animal species were used in this study, which differed from the previous report.

In chronic toxicity testing, there are inferences about the long-term effect of a test substance in animals, and they may be extrapolated to the test substance’s human safety. The duration of the exposure period should be repeated oral administration of a test substance for 9 to 12 months. During the experiment, the animals are monitored for general physiological functions, behavioral variations, and alterations in biochemical parameters. At the end of the experiment, internal organs are collected from the animals and subjected to histopathological analyses [14,15].

In the present study, PCT was continuously fed to the female and male rats for 270 days at doses of 13, 130, and 1300 mg/kg. To assess potential PCT toxicity, animals’ symptoms and external characteristics were recorded throughout the experiment. In PCT-treated rats, the results revealed no symptoms, changes in animal behavior, or toxic signs. Then, changes in body weight and internal organ weight were observed, as body weight and internal organ weight are two main indicators used to determine the onset of toxicity caused by a test substance. As a result, although the body weight of the PCT (13 mg/kg)-treated female group showed gains, the difference was slight and was still within normal limits. The internal organs, including the brain, lungs, heart, liver, spleen, adrenal glands, and sex organs, were weighed and explored for histopathology. In this study, we found that the internal organ weights of some groups of female and male rats increased and decreased; the differences were slight and remained within the normal range. In PCT-treated rats, the histopathological examination of these organs showed no change.

The hematological values, the difference in white blood cell count, and the biochemical values were evaluated. The detection of abnormalities in various systems of experimental animals was based on the hematological values and the difference in white blood cell count. In addition, the liver and kidneys are organs that perform normal metabolism and excretion, making it simple for toxins to destroy these organs. Therefore, the liver’s and kidneys’ efficiency can be assessed using blood chemistry values, which indicate the physiological conditions of important organs or the pathological characteristics of experimental animals [15]. In this study, it was found that female rats that received PCT (130 mg/kg) had decreased PLT, whereas the group that received PCT (1300 mg/kg) had an increased HCT value and was different from the control group. As for WBCs, it was found that female rats that received PCT (13 mg/kg) had decreased MONO counts, while the group that received PCT (130 mg/kg) had decreased LYMP and MONO counts and were different from the control group. However, when the hematology and WBC values were compared with the standard values, they were found to be within the standard range [16,17]. This study indicates that PCT did not affect the characteristics and the amount of blood cells, including blood components.

Clinical blood chemistry can be used to assess the efficiency of important body organs, including the pancreas, kidneys, and liver. The GLU is a preliminary test of pancreatic function. BUN and CRE levels are used to indicate kidney function and efficiency. The values of TP, ALB, T-BIL, D-BIL, ALP, ALT, and AST indicate liver function and pathology. Insulin, the main hormone produced by pancreatic beta cells, is used to control blood sugar levels. It works with glucagon and other hormones, such as growth hormone, cortisol, and catecholamine. Insulin is the only hormone that can reduce blood sugar levels. Other hormones have the opposite effect of promoting sugar production. Therefore, if blood sugar levels increase due to exposure to the test substance, it may be the result of the test substance destroying pancreatic cells. The study’s findings revealed that male rats receiving PCT at doses of 13 and 130 mg/kg had lower blood sugar levels. However, these values were still within the standard range [18].

The study of abnormalities of tissues and organs in both sexes of all groups of rats compared with the control group is the final step and provides important evidence to indicate whether the test substance can cause chronic toxicity. The gross pathological and histopathological examinations of various organs, including the brain, lung, heart, liver, spleen, adrenal glands, kidneys, ovaries, uterus, and testes, found no lesions or abnormal appearances, sizes, shapes, or colors when compared with the control group, especially in the important internal organs, namely the liver and kidneys. Furthermore, no physical or behavioral abnormalities were found that indicated any physiological abnormalities in the experimental animal body, including pathological abnormalities in various internal organs.

The chronic study also revealed several sex-dependent differences that merit explicit comment. In male rats, fasting glucose was modestly reduced at the 13 and 130 mg/kg doses, whereas female rats showed no such change; conversely, the scattered hematological shifts (platelet count and lymphocyte and monocyte fractions) were more apparent in females, and a lower testis weight was recorded in the lowest-dose (13 mg/kg) male group. Several features argue against these being toxicologically meaningful treatment-related effects. The changes were generally small, were not dose-ordered (for example, the testis-weight reduction occurred at the lowest but not at the intermediate or highest dose, and the glucose reductions did not increase with dose), and all values remained within the published reference ranges for Sprague-Dawley rats. Sex-related variations in baseline glucose handling, leucocyte populations and organ weights are well documented in this strain and are the most parsimonious explanation for the present observations, a conclusion reinforced by the absence of any corresponding gross or histopathological abnormality in the liver, kidneys, pancreas or reproductive organs of either sex. We nonetheless report these sex differences explicitly, because the present design was not powered to characterize sex as a biological variable; an adequately powered study incorporating reproductive and endocrine endpoints would be required before any sex-specific effect of PCT on glucose homeostasis or testicular weight could be confirmed or excluded.

Allergic rhinitis (AR) is a type 2 inflammatory disease characterized by allergen-specific immunoglobulin E (IgE) production and infiltration of inflammatory cells such as basophils, eosinophils, T lymphocytes, and T helper 2 (Th2)-derived cytokines [19,20]. It is well known that there are several types of T helper cells involved in AR, such as T helper 1 (Th1) cells, T helper 2 (Th2) cells, and regulatory T cells (Treg). Th1 cells associated with immune responses produce interleukin (IL)-12 and interferon (IFN)-γ, which are pro-inflammatory cytokines. According to [21], Th1 cytokines can inhibit Th2 cytokines, promote Th1 cell differentiation, and act as a powerful T cell function stimulator. Th2 cells associated with immune responses produce IL-4, IL-5, and IL-13. Th2 cytokines can promote Th2 cell differentiation, IgE production, eosinophil activation and recruitment, and mucus production [22]. Another subtype of T cells, regulatory T cells (Treg), produces IL-10 and transforming growth factor (TGF)-β. Treg cytokines can suppress Th1, Th2, and Th17 cytokine expression, reduce eosinophils, mast cells, and basophils, inhibit inflammatory cell migration to tissues, and induce IgE class switching to IgG4 [23]. Mice are established animal models for effective treatments for allergic rhinitis. BALB/c mice are generally preferred for studies on allergic immune responses because they have been shown to provide more robust allergic rhinitis responses than other mouse strains [24,25]. Furthermore, ovalbumin (OVA) is the universal reagent for experimental allergen use and is often given in combination with aluminum hydroxide gel (alum) adjuvant, which is widely used as an allergen to induce allergic rhinitis in mouse models because it does not evoke additional innate, non-AR immune responses to specific microbe-associated molecular patterns, which could greatly complicate any analysis of AR [24,26].

Previously, PCT has been reported to have anti-allergic activity. The pharmacological study of PCT showed that the ethanolic extract of PCT had a potent inhibitory effect on the release of β-hexosaminidase from RBL-2H3 cell lines with an IC_50_ value of 12.97 ± 1.92 μg/mL [10]. Clinical trials on PCT powder for allergic rhinitis treatment involving twenty-four patients who received a PCT remedy (3000 mg/day) for six weeks showed that the patients had significantly improved nasal symptoms and a better quality of life [27]. Furthermore, a powdered PCT remedy (4000 mg/day) taken for seven days decreased the overall nasal symptoms of allergic rhinitis and upper respiratory tract infections in patients but was not different from loratadine, and the treatment period was shorter [28]. However, the in vitro anti-allergic activity of plant ingredients in the PCT remedy and the anti-allergic activity of the PCT remedy in experimental animals have not yet been studied. We conducted experiments to elucidate the anti-allergic activities of the PCT remedy both in vitro and in vivo using IgE-stimulated RBL-2H3 cells and OVA-sensitized AR in an animal model.

In our in vitro study, the anti-allergic activity of the PCT remedy and its plant ingredients was tested using a β-hexosaminidase release assay in RBL-2H3 cells, a widely used model for mast cell degranulation studies. The enzyme β-hexosaminidase, which is released in parallel with histamine, is commonly used as a marker for degranulation in RBL-2H3 cells [29]. Our results demonstrated that the PCT remedy extract inhibited β-hexosaminidase release from degranulation in antigen-induced IgE-sensitized RBL-2H3 cells. The 95% ethanolic extract of the PCT remedy showed the most potent activity on β-hexosaminidase release, with an IC_50_ value of 13.18 ± 2.67 µg/mL, 1.7-fold more potent than the positive control drug, CPM (IC_50_ = 22.12 ± 1.07 µg/mL). Moreover, *C*. *excavata* (CEE) ethanolic extract revealed the highest activity on β-hexosaminidase release, with a potency 3.2 times that of CPM, followed by *P*. *retrofractum* and *P*. *indica*, with IC_50_ values of 9.97 ± 2.15 and 10.88 ± 0.66 µg/mL and potencies 2.2 and 2.0 times that of CPM, respectively. These results correlated with a previously reported study that the ethanolic extracts of *P*. *retrofractum* and *P*. *indica* and its active compounds, such as piperine from *P*. *retrofractum* and plumbagin from *P*. *indica*, had highly in vitro anti-allergic activity, with IC_50_ values of 15.82 ± 2.00, 13.31 ± 1.78, 14.14 ± 1.46, and 4.03 ± 0.78 µg/mL, respectively [30]. In addition, we found that the plant ingredients attributed to the hot and spicy flavor contained in the PCT remedy, i.e., *M*. *fragrans* (Nutmeg) (MFE(N)), *P*. *nigrum* (PNE), *Z*. *officinale* (ZOE), *L*. *sibiricus* (LSE(K)), *A*. *lancea* (ALE), *M*. *fragrans* (Mace) (MFE(M)), *S*. *aromaticum* (SAE), and *F*. *vulgare* (FVE), also possessed highly active inhibition of β-hexosaminidase release, and they were 1.8-, 1.8-, 1.5-, 1.4-, 1.2-, 1.2-, 1.1-, and 1.0-fold more potent than CPM, respectively. Previous studies reported that the extracts obtained from nutmeg and mace of *M*. *fragrans* exhibited strong anti-allergy activity, with IC_50_ values of 20.90 ± 1.03 and 12.95 ± 0.89 µg/mL, respectively [31]. The ethanolic extract of *P*. *nigrum* and piperine, a major substance in the fruit, possessed an inhibitory effect on antigen-induced degranulation in RBL-2H3 cells, with IC_50_ values of 14.0 and 16.0 µg/mL, respectively [32]. In this study, *Z*. *officinale* expressed apparently strong anti-allergic activity, similar to our previous report that the rhizome of *Z*. *officinale* was highly active using the same method (IC_50_ = 12.93 ± 1.28 µg/mL) [30]. In contrast, Tewtrakul and Subhadhirasakul [33] reported that *Z*. *officinale* ethanolic extract showed moderate anti-allergic activity (IC_50_ = 40.3 µg/mL) compared to our study, which may reflect various plant collecting seasons, geographic locations, and extract preparation techniques.

In our in vivo study, anti-allergic rhinitis activity was evaluated in OVA-sensitized and OVA-challenged mice treated with different dosages of PCT remedy extract. The results showed that PCT improved allergic rhinitis mainly by inhibiting the infiltration of inflammatory cells in the nasal mucosa and regulating cytokine levels in the nasal mucosa, rather than by lowering the serum levels of OVA-specific immunoglobulins. Immunoglobulin antibodies such as IgE, IgG1, and IgG2a are controlled by cytokines from T helper cells and are associated with B-cell immune responses [34]. The levels of serum OVA-specific IgE (a marker of Th2 cells), IgG1 (a marker of Th2 cells), and IgG2a (a marker of Th1 cells) were significantly higher in the control group than in the sham group, confirming successful allergic sensitization. However, treatment with PCT (75, 150, and 300 mg/kg) and loratadine (10 mg/kg) did not significantly reduce the serum levels of OVA-specific IgE or IgG2a compared with the control group, and OVA-specific IgG1 was likewise not reduced; in fact, the PCT (300 mg/kg) group showed a slight but significant increase in OVA-specific IgG1 relative to the control group. These results suggest that, in this model, the anti-allergic effect of PCT was not mediated by a reduction in circulating OVA-specific immunoglobulins but was instead associated with the local responses in the nasal mucosa described below. The dissociation between the local downregulation of IL-5 and IL-13 and the unchanged or increased systemic OVA-specific immunoglobulins is consistent with compartmentalized regulation of mucosal and systemic immunity, in which tissue cytokine responses and circulating antibody titers need not change in parallel. It is also compatible with the different time courses of the two readouts, since class-switched antibody is comparatively stable in the circulation, whereas tissue cytokine expression responds more rapidly to treatment. Taken together, these observations indicate that PCT acts predominantly on the effector phase of the allergic response in the nasal mucosa rather than on the upstream sensitization and antibody-production phase, which would account for a local anti-inflammatory effect occurring without a reduction in, and even alongside a rise in, OVA-specific IgG1.

During allergic reactions, numerous inflammatory cells, particularly eosinophils, infiltrate the nasal mucosa, which is triggered by Th2 cells [35]. Moreover, hypersecretory mucus production is a characteristic feature of mucosal inflammation in AR, which is produced by goblet cells [36]. Mast cells are also associated with inflammatory sites in allergic disorders and are located in mucosal epithelial surfaces and connective tissues [35]. Treatment with PCT (75, 150, and 300 mg/kg) and loratadine (10 mg/kg) decreased the number of inflammatory cells infiltrating the nasal mucosa and significantly reduced mast cell infiltration and mucus production in nasal tissues when compared with the control group. However, PCT at a concentration of 75 mg/kg and loratadine at a concentration of 10 mg/kg were not significant in reducing mucus production. These results suggest that PCT may mitigate AR symptoms by suppressing Th2-related allergic reactions.

Our findings demonstrate that treatment with PCT (75, 150, and 300 mg/kg) resulted in significant alleviation of Th2-cytokine expression levels, including IL-5 and IL-13, in the nasal mucosa of mice. This reduction in Th2 cytokine expression may contribute to the suppression of inflammatory cell infiltration and inhibition of mucus production in the treated mice. Furthermore, PCT treatment produced only a non-significant trend toward increased TGF-β mRNA expression, so the anti-allergic effect observed in this model was not associated with a statistically significant change in this Treg-associated cytokine. In addition, IL-12b mRNA expression, a Th1 cytokine, was reduced in the nasal tissues of allergic mice following PCT treatment at all doses. The reason for the reduction in IL-12b profile remains unclear, but it may be due to compensatory suppression of other cytokines. Because a reduction in this Th1-associated cytokine does not fit neatly within a simple Th1/Th2 framework, we interpret it cautiously and flag it as a limitation that warrants dedicated mechanistic investigation. One possibility is that the concurrent reduction of this Th1-associated cytokine reflects a broad immunomodulatory action of the remedy across several cytokine pathways rather than a selective effect on Th2 responses, which would be in keeping with the polyherbal composition of PCT and its many reported anti-inflammatory constituents. Furthermore, various plant ingredients used in PCT have been reported to have anti-allergic properties in animal studies. For instance, a 70% ethanolic extract of *A*. *dahurica* (50 and 100 mg/kg) was found to significantly downregulate IL-4, IL-5, and TNF-α levels in BALF and reduce inflammatory cells in a mouse model, demonstrating its anti-allergic potential [37]. The main active constituent of *A*. *dahurica*, imperatorin (IMP), was also found to inhibit mas-related G protein-coupled receptor X2 (MRGPRX2), demonstrating a novel potential in treating allergic asthma [38]. Macelignan was isolated from *M*. *fragrans* and showed reduction of IL-4 and IL-17 production by inhibiting CD4+T cells in a fungal protease mixed with chicken egg ovalbumin-induced murine asthma model [39]. The ethanolic extract of *N*. *sativa* inhibited histamine release from peritoneal Wistar rat mast cells and was reported to have potential in treating allergic asthma in animal models [40,41,42]. Thymoquinone (TQ), an active compound of *N*. *sativa*, was also found to reduce IL-4 and OVA-specific IgE production, as well as TNF-α and IL-1β expression, eosinophil infiltration, and edema in the nasal mucosa in a rat model of allergic rhinitis [43]. TQ also reduced blood cytokines and changed the histopathology in lung lavage fluid in a guinea pig model of asthma [44]. *P*. *nigrum* fruit extract was found to suppress allergic responses by inhibiting inflammatory cell accumulation, improving nasal histopathology, and inhibiting NFκBp65 and STAT3 signaling activation. It also alleviated allergic asthma by inhibiting Th2/Th17 responses and mast cell activation and improving epithelial barrier dysfunction by increasing Nrf2/HO-1 signaling activation [2,45,46]. Moreover, piperine isolated from *P*. *nigrum* significantly enhanced sneezing, rubbing, and redness. It also reduced histamine, NO levels, IL-6, IL-1β, and IgE expression, and inflammatory cell infiltration [47]. Another study indicated that piperine also attenuated eosinophil infiltration and decreased airway hyperresponsiveness by suppressing T cell activity and Th2 cytokine production in an OVA-induced asthma model [48]. Active compounds isolated from *Z*. *officinale*, such as 6-gingerol and 6-shogaol, effectively suppressed the allergic response by inhibiting inflammatory cell infiltration, airway mucus production, and Th2 cell-mediated inflammatory cytokine production in the lung tissues of an asthmatic mouse model [49]. *Z*. *officinale*, 6-gingerol, 6-shogaol, and zingerone have also been reported to ameliorate allergic rhinitis and asthma in murine models [50,51,52]. Therefore, the combination of these ingredients in PCT could lead to a synergistic anti-allergic effect. These ingredients are combined in the PCT remedy according to traditional Thai medicine principles of remedy compatibility, in which the predominant hot and spicy constituents are intended to act together to disperse wind and relieve respiratory and allergic symptoms. The convergent anti-allergic actions of the individual ingredients documented above provide a pharmacological rationale for this traditional pairing and suggest that the remedy acts through additive or synergistic modulation of mast cell degranulation and Th2 cytokine signaling rather than through any single dominant constituent.

Several limitations of the present study should be acknowledged. First, the evidence is derived entirely from rodent models, and the anti-allergic and safety findings will require confirmation in non-rodent species and, ultimately, in controlled clinical studies before they can be extrapolated to human use. Second, PCT is a twenty-three-plant polyherbal extract; the present work establishes activity at the level of the remedy and its individual ingredients but cannot yet attribute the effects to specific compounds or pathways. Third, the in vivo allergic rhinitis experiment used six animals per group, which provides limited precision for effect-size estimation given the variance observed in the histological scores, and the acute toxicity study, in line with OECD Test Guideline 420, was conducted in female rats only; sex-balanced and larger cohorts would strengthen both the safety and the efficacy conclusions. Fourth, in the in vitro assay, only IC_50_ values were derived; however, parallel RBL-2H3 viability testing at the tested concentrations was not performed, so a contribution of cytotoxicity to the apparent inhibition of degranulation, particularly for the most potent extracts such as *C*. *excavata*, cannot be formally excluded. Finally, the large number of hematological and biochemical parameters compared in the toxicity studies, analyzed without correction for multiple comparisons, means that the isolated, non-dose-related differences reaching nominal significance should be regarded as hypothesis-generating rather than definitive. Future work should focus on bioassay-guided identification of the active constituents and their molecular targets, dedicated cytotoxicity and mechanistic counter-screens, and adequately powered, sex-balanced in vivo studies.

## 4. Materials and Methods

### 4.1. Chemicals and Reagents

Commercial-grade 95% ethanol was purchased from C.M.J. Anchor Co., Ltd. (Bangplee, Samutprakarn, Thailand). Anti-DNP IgE (Monoclonal Anti-DNP), anti-dinitrophenylated bovine serum albumin (DNP-BSA), *p*-nitrophenyl-*N*-acetyl-β-D-glucosaminide (PNAG), albumin bovine fraction V power, D-(+)-glucose, paraformaldehyde, bovine serum albumin (BSA), gelatin type B from bovine skin and albumin from chicken egg white grade V (OVA), loratadine, and paraformaldehyde were purchased from Sigma-Aldrich (St. Louis, MO, USA). Calcium chloride dihydrate, citric acid monohydrate, magnesium chloride 6H_2_O, potassium chloride, sodium carbonate and sodium bicarbonate were purchased from Merck (Darmstadt, Germany). Tween-80, imject alum adjuvant and the RevertAid First Stand cDNA Synthesis Kit were purchased from Thermo Scientific (Rockford, IL, USA). Fetal bovine serum (FBS), phosphate-buffered saline (PBS) and trypsin-EDTA were purchased from Gibco Life Technologies Limited (Paisley, UK). Minimum essential medium (MEM), penicillin-streptomycin (P/S) and trypan blue were purchased from Gibco Life Technologies Corporation (Grand Island, NY, USA). Piperazine-*N*,*N*′-bis(2-ethanesulfonic acid) (PIPES) was purchased from Amresco (Cleveland, OH, USA). Sodium chloride, sodium hydroxide and sucrose were purchased from Univar (Sydney, NSW, Australia). Goat-anti-mouse IgG1-biotin; Cat.no. 1070-08, rat-anti-mouse IgE-biotin; Cat.no. 1130-08, rat-anti-mouse IgG2a-biotin; Cat.no. 1155-08 and streptavidin-horseradish peroxidase (HRP) conjugate; Cat.no. 7100-05 were purchased from Southern Biotech (Birmingham, AL, USA). ABTS peroxidase substrate was purchased from KPL SeraCare Life Sciences (Milford, MA, USA). Brilliant II SYBR Green QPCR Master Mix was purchased from Agilent Technologies (Santa Clara, CA, USA). Total RNA mini kit (tissue) was purchased from Geneaid Biotech Ltd. (Shijr, New Taipei, Taiwan). RNA*later* RNA stabilization reagent was purchased from QIAGEN (Hilden, Germany).

### 4.2. Plant Materials

The Prabchompoothaweep (PCT) remedy consists of a mixture of twenty-three different plants, each in specific proportions. The plants used are as follows: *Acanthus ebracteatus*, *Amomum testaceum*, *Amorphophallus saraburiensis*, *Angelica dahurica*, *Ardisia polycephala*, *Atractylodes lancea*, *Cinnamomum camphora*, *Clausena excavata*, *Enhalus acoroides*, *Foeniculum vulgare*, *Leonurus sibiricus*, *Lepidium sativum*, *Myristica fragrans* (Nutmeg), *Myristica fragrans* (Mace), *Nigella sativa*, *Piper retrofractum*, *Piper nigrum*, *Plumbago indica*, *Syzygium aromaticum, Terminalia arjuna*, *Terminalia chebula*, and *Zingiber officinale*. All the plants used in the PCT remedy were purchased from Charoensuk Osot Pharmacy in Bangkok, Thailand, during November and December 2018. Authentication of plant materials was by comparison with specimens deposited at the herbarium of the Southern Center of Thai Medicinal Plants at the Faculty of Pharmaceutical Sciences, Prince of Songkla University, Songkhla, Thailand. The voucher specimens and proportions of each plant used are shown in Table S1.

### 4.3. Animals

For toxicity testing, both male and female Sprague-Dawley rats weighing between 180 and 200 g were purchased from Nomura Siam International Co., Ltd. (Bangkok, Thailand). The rats were housed (2–3 rats per cage) and maintained in an animal room at a controlled temperature of 25 ± 1 °C, a relative humidity of 60%, and a 12 h light/dark cycle. *Ad libitum* access to chow and water was provided to the rats during the study. All rats were acclimatized for one week before the start of the experiment. Rats were not fasted prior to terminal blood collection for biochemical parameter measurements. The Animal Ethics Committee, Faculty of Medicine, Chiang Mai University (Chiang Mai, Thailand) approved all animal experiments (protocol number 42/2561, approved on 12 March 2019).

For anti-allergic activity: male BALB/cAJcl mice, weighing between 20 and 30 g and aged 5–7 weeks, were procured from Nomura Siam International Co., Ltd. (Bangkok, Thailand). Throughout the study, mice (3 mice per cage) were kept in a controlled environment with a temperature of 22 ± 1 °C, a relative humidity of 30–70%, and a 12 h light/dark cycle. *Ad libitum* access to chow and water was provided to the mice during the study. Before the start of the experiment, all mice were acclimatized for one week. The Institutional Care and Use Committee of Thammasat University (Pathumthani, Thailand) approved all animal experiments (protocol number 032/2561, approved on 18 April 2019). The design, conduct and reporting of the in vivo experiments followed the ARRIVE 2.0 guidelines.

### 4.4. Preparation of Sample Extraction

Plant materials were cleaned, cut into small pieces, oven-dried at 50 °C, and ground until they passed through a 4-mesh sieve into a fine powder. The powdered plants were mixed in the proportions listed in Table S1 to make the PCT remedy. Each powdered plant material (0.5 kg) and the mixed remedy (10 kg) were macerated with 95% ethanol 2 L and 40 L, respectively, for 72 h at room temperature, filtered through Whatman filter paper No.1, and then macerated twice more. The filtered extract was concentrated using a rotary evaporator (45 °C) and then dried in a hot air oven at 45 °C. The extract was kept in the refrigerator at −20 °C until used.

#### Quantitation of PCT Compound Using HPLC

The HPLC method was slightly modified following a previous report [53]. The analysis was carried out using an Agilent LC1200 with a photodiode array detector (DAD) and an autosampler. The analytical column was a reversed-phase C18 column (Phenomenex Luna C18, size 250 mm × 4.6 mm, 5 µm) with a guard cartridge (Phenomenex (C18), size 4 mm × 3.0 mm) with a guard holder. The mobile phase was composed of water (A): acetonitrile (B). The gradient elution profile was as follows: 0–25 min, (A:B; 60%:40%), 25–50 min, (A:B; 50%:50%), 50–60 min, (A:B; 5%:95%), 60–65 min, (A:B; 0%:100%), 65–70 min, (A:B; 60%:40%), and 70 min, (A;B; 60%:40%), respectively. The analysis was performed at room temperature with a wavelength of 340 nm and a flow rate of 1.0 mL/min. The sample solutions of PCT were prepared at a concentration of 10 mg/mL in acetonitrile and sonicated for 15 min, filtered with a 0.45 µm membrane filter, and injected at a volume of 10 µL. Piperine was used as a reference marker, with a concentration of 200, 100, 50, 25, and 12.5 µg/mL in acetonitrile. The quantitative HPLC analysis was conducted based on the peak area of the component.

### 4.5. Acute Toxicity Test

In the sighting study, female Sprague-Dawley rats were given an initial dose of 2000 mg/kg of extract according to OECD Test Guideline 420 (Annex 2-Flow chart of the sighting study). However, no toxicity was noted. Thus, the acute toxicity study’s upper limit was set at 5000 mg/kg of PCT as the main study starting dose [54]. We acknowledge that the standard limit dose under OECD Test Guideline 420 is 2000 mg/kg and that a 5000 mg/kg dose is reserved for situations in which a wide margin of human safety must be demonstrated. PCT is a traditional polyherbal remedy administered to patients at high daily doses (of the order of 3000 to 4000 mg/day), so confirming the absence of acute toxicity at 5000 mg/kg was considered necessary to define an adequate safety margin for clinical use. To make the study flow explicit, the sighting study was performed in two female rats (*n* = 2) at 2000 mg/kg and, because no toxicity was observed, the main study then compared a distilled-water control group with a 5000 mg/kg group, each comprising five female rats (*n* = 5 per group). The female rats were divided into two groups of five rats each. Group 1 (control group) rats were orally administered distilled water (2 mL/kg), and Group 2 (test group) rats received PCT at a dose of 5000 mg/kg in 2 mL/kg distilled water. The general behavior of rats and signs of toxicity such as abnormal breathing, lethargy, vomiting, muscle spasticity, and water diarrhea were observed within the first 6 h after PCT administration and every day until the end of 14 days. The body weights were monitored on days 1, 7 and 14. The mortality of the rats was recorded. At the end of the study, all surviving rats were sacrificed, and the internal organs, including the brain, heart, lungs, liver, spleen, kidneys, adrenal glands, ovaries, and uterus, were removed, weighed, and histopathologically examined. The organs were preserved in 10% formaldehyde for histopathological examination.

### 4.6. Chronic Toxicity Test

The chronic toxicity study was carried out according to the OECD Test Guideline 452 [55]. Within each sex, animals were allocated to groups by simple randomization using a computer-generated sequence of random numbers, and cages and treatments were coded so that group identity was concealed during dosing and routine handling. The rats were randomly divided by sex into six groups; each main group comprised ten males and ten females (*n* = 10 per sex), and each satellite group (Groups 2 and 6) comprised five males and five females (*n* = 5 per sex), as follows: Group 1, the control group, received distilled water (2 mL/kg/day); Group 2, the satellite group of Group 1, received distilled water (2 mL/kg/day); Group 3, the treatment group, received 13 mg/kg/day of PCT; Group 4, the treatment group, received 130 mg/kg/day of PCT; Group 5, the treatment group, received 1300 mg/kg/day of PCT; Group 6, the satellite group of Group 5, received 1300 mg/kg/day of PCT. Both Group 2 and Group 6, after receiving PCT, were observed for 28 days with no PCT administered before being sacrificed. In the treatment groups, all rats received oral administration of PCT every day for 270 days. During the study period, the behavioral changes, signs of toxicity, physiological parameters, and body weights of the rats were monitored. Body weight was recorded daily, and food and water intake were monitored daily throughout the 270-day dosing period. An autopsy was carried out to confirm the death of the animal. At the end of 270 days, blood samples were collected from all rats in Groups 1, 3, 4, and 5 for hematology and biochemistry evaluation. After that, all rats were sacrificed, and their internal organs, including the brain, heart, lungs, liver, spleen, kidneys, adrenal glands, and sex organs, were removed, weighed, and then histopathologically examined. The organs were preserved in 10% formaldehyde for histopathological examination. Hematological and biochemical analyses were performed using standard analytical techniques. For hematological analysis, red blood cell (RBC) count, hemoglobin (HB), hematocrit (HCT), mean corpuscular volume (MCV), mean corpuscular hemoglobin (MCH), mean corpuscular hemoglobin concentration (MCHC), platelet count (PLT), and white blood cell (WBC) count, including neutrophil (PMN), lymphocyte (LYMP), monocyte (MONO), eosinophil (EOS), and basophil (BASO) counts, were measured. For biochemical analysis, glucose (GLU), blood urea nitrogen (BUN), creatinine (CRE), total protein (TP), albumin (ALB), total bilirubin (T-BIL), direct bilirubin (D-BIL), aspartate aminotransferase (AST), alanine aminotransferase (ALT), and alkaline phosphatase (ALP) levels were measured. In both Groups 2 and 6, all rats were observed for an additional 28 days before blood samples were collected and necropsies were performed [15,55].

### 4.7. In Vitro Anti-Allergic Activity on β-Hexosaminidase Release from RBL-2H3 Cell Lines

Rat basophilic leukemia cell line (RBL-2H3) was purchased from American Type Culture Collection (ATCC^®^ CRL-2256^TM^, VA, USA). This cell line corresponds to Cellosaurus accession CVCL_0591 (RRID: CVCL_0591). The passage number of RBL-2H3 cells used in these experiments was 10 to 15 passages, and cells were used within a limited passage range to minimize phenotypic drift. The RBL-2H3 cells were cultured in MEM supplemented with 15% heat-inactivated FBS, 10,000 units/mL penicillin, and 10,000 µg/mL streptomycin. The cells were maintained in an incubator at 37 °C in a humidified 5% CO_2_/95% air atmosphere. Inhibitory effects on the release of β-hexosaminidase from RBL-2H3 cells were evaluated according to a previously described method [56]. The cells were seeded at a concentration of 2 × 10^5^ cells/400 µL/well in 24-well plates and allowed to adhere for 2 h at 37 °C in 5% CO_2_. The cells were then sensitized with anti-dinitrophenyl-immunoglobulin E (anti-DNP IgE) (0.45 µg/mL) and incubated at 37 °C in 5% CO_2_ for 24 h. After that, the cells were washed with 400 µL of Siraganian buffer [buffer A; consisting of 119 mM NaCl, 5 mM KCl, 5.6 mM D-(+)-glucose, 0.4 mM MgCl_2_.6H_2_O, 1 mM CaCl_2_.2H_2_O, 25 mM piperazine-*N*,*N*′-bis(2-ethanesulfonic acid) (PIPES), 0.1% bovine serum albumin (BSA) and 40 mM NaOH, pH 7.2], and incubated in 160 µL of Siraganian buffer (buffer A) for an additional 10 min at 37 °C in 5% CO_2_. Various concentrations of test samples (20 µL) were added to each well and incubated for 10 min, followed by the addition of 20 µL of antigen (DNP-BSA, final concentration 10 µg/mL) for 20 min at 37 °C in 5% CO_2_ to stimulate the cells into producing allergic reactions. Aliquots (50 µL) of supernatant were transferred into 96-well plates and incubated with 50 µL of substrate (PNAG) (1 mM *p*-nitrophenyl-*N*-acetyl-β-D-glucosaminide) in 0.1 M citrate buffer (pH 4.5) at 37 °C in 5% CO_2_ for 2 h. The reaction was stopped by adding 200 µL of a stop solution (0.1 M Na_2_CO_3_/NaHCO_3_, pH 10.0). The optical density was measured using a microplate reader at 405 nm (OD_405 nm_). The test sample was dissolved in dimethyl sulfoxide (DMSO), and the solution was added to Siraganian buffer (final DMSO concentration was 0.1%).

The inhibition (%) of release of β-hexosaminidase by the test sample was calculated by the following equation, and IC_50_ values were calculated using the Prism program.

Inhibition (%) = [1 − (T − B − N)/(C − B − N)] × 100

where Control (C): DNP-BSA (+), test sample (−); Test (T): DNP-BSA (+), test sample (+); Blank (B): DNP-BSA (−), test sample (+); Normal (N): DNP-BSA (−), test sample (−)

### 4.8. In Vivo Anti-Allergic Activity Testing

The PCT remedy, which showed highly potent in vitro anti-allergic activity, was then tested for in vivo anti-allergic activity.

#### 4.8.1. Ovalbumin (OVA)-Induced Allergic Rhinitis (AR) Mouse Model

The OVA-induced AR mouse model was established with modifications following a previous report [57]. Animals were allocated to groups by simple randomization using a computer-generated sequence of random numbers, and cages and treatments were coded. Mice were randomly distributed into six groups, with six mice in each group. Mice in Group 1 (sham group) were treated with PBS, without sensitization or challenge. Mice in Groups 2–6 were sensitized on days 0, 7, and 14 by intraperitoneal (i.p.) injection with 25 µg/mL of OVA in phosphate-buffered saline (PBS) mixed with 1 mg of alum adjuvant in a total volume of 200 µL. After one week, mice in Groups 2–6 were challenged three times a week for four weeks by intranasal (i.n.) instillation with 500 µg/mL of OVA in PBS into each nostril (10 µL per nostril). One hour after the challenge, mice in Group 2 (solvent control group) were treated by oral gavage with 5% Tween-80; mice in Group 3 were treated with 10 mg/kg of loratadine, while mice in Groups 4–6 were administered with 75, 150, and 300 mg/kg of PCT, respectively. On day 45, all mice were euthanized by intraperitoneal (i.p.) injection with a thiopental overdose, and their blood was collected individually from cardiac puncture and centrifuged at 3000 rpm for 10 min at 4 °C to obtain serum for measuring the levels of OVA-specific serum IgE, IgG1, and IgG2a antibodies by indirect enzyme-linked immunosorbent assay (indirect ELISA). Nasal tissue was then removed from the body for cytokine study and histopathologic investigation.

#### 4.8.2. Indirect ELISA for Measuring OVA-Specific IgE, IgG1, and IgG2a in Mouse Serum

To measure OVA-specific IgE, IgG1, and IgG2a in mouse serum, an indirect ELISA was performed [58]. A microtiter ELISA plate was coated with 1 μg of OVA in 100 μL of carbonate-bicarbonate buffer (pH 9.6) and incubated at 37 °C until dried. The plate was washed with PBST and blocked with a 1% BSA solution in PBS. After incubating for 1 h in a moist chamber, the plate was washed again. Mouse serum, diluted 1:4 for IgE and 1:1000 for IgG1 and IgG2a in a diluent containing 0.2% gelatin and 0.2% BSA in PBS, was added to the antigen-coated plate and incubated at 37 °C for 1 h. The plate was stored at 4 °C overnight. Rat-anti-mouse IgE-biotin, goat-anti-mouse IgG1-biotin, and rat-anti-mouse IgG2a-biotin were added, followed by incubation at 37 °C for 1 h. The plate was washed with PBST, and streptavidin–horseradish peroxidase conjugate was added and incubated at 37 °C for 1 h. After washing with PBST, ABST substrate was added and left in the dark at room temperature for 20 min for IgE and 5 min for IgG1 and IgG2a. Finally, the reaction was stopped by adding 100 μL of a stop solution (1% SDS in distilled water). The optical density (OD) was measured using a microplate reader at 405 nm.

#### 4.8.3. Histopathological Analysis of the Nasal Tissue

The right side of the head of each mouse was fixed with 5% paraformaldehyde and 4% sucrose in PBS. The nasal tissue was then embedded in paraffin, sectioned to 5 µm, and placed on a slide. Hematoxylin and eosin (H&E), toluidine blue (TB), and Periodic acid-Schiff (PAS) staining were used to analyze inflammatory cells, mast cell granules, and mucus production, respectively. All slides were observed under a microscope at 400× magnification. Histological grading of inflammatory cell infiltration, mucus production and mast cell numbers was performed by an assessor who was blinded to the treatment allocation, and the same blinded coding was applied to the toxicity histopathology. Inflammatory cells that infiltrated into the epithelium were graded on a scale from 1 to 4 (Table S5), and the intensity of magenta color in PAS-stained mucus glands was graded on a scale from 1 to 3 (Table S5). The number of mast cells was counted in 10 microscopic fields per section per mouse.

#### 4.8.4. Study of Cytokines in Mouse Nasal Tissue

The cytokine gene expression was determined by quantitative real-time PCR (qPCR) [59]. The left side of the head of each mouse was preserved using RNA*later* RNA stabilization reagent and stored at −80 °C. Total RNA was extracted from the nasal tissue using the Total RNA mini kit (tissue) according to the manufacturer’s protocols. RNA quality and integrity were assessed by A260/A280 ratio prior to cDNA synthesis. Complementary DNA (cDNA) was synthesized using the RevertAid First Strand cDNA Synthesis Kit according to the manufacturer’s instructions. The cDNA was used as a template for quantification of housekeeping gene (β-actin) and cytokine gene expression (mRNA), which included IL-5, IL-13, IL-12b (p40) and TGF-β. The sequences of the oligonucleotide primers used for gene amplification are listed in Table S6 [60]. The PCR mixture contained 1 µL of cDNA and 100 nM of each forward and reverse PCR primer in Brilliant II SYBR Green QPCR Master Mix. The reactions were run using the StepOneTM Real-Time PCR System for 40 cycles. StepOneTM software version 2.3 was used for data analysis. Thermal cycling conditions included an initial denaturation at 95 °C for 10 min, followed by 40 cycles of amplification at 95 °C for 30 s, annealing at 60 °C for 1 min, and extension at 72 °C for 30 s. Each target cytokine mRNA level was normalized versus the housekeeping β-actin gene. Reactions were performed in duplicate, and relative fold changes in cytokine mRNA expression were calculated using the comparative Ct (2^−ΔΔCt^) method with β-actin as the reference gene.

### 4.9. Statistical Analysis

In the acute and chronic toxicity studies, the data are presented as mean ± standard error of the mean (SEM). The statistical significance was calculated by one-way ANOVA, followed by a post hoc least-significant difference (LSD) test. In the in vitro and in vivo anti-allergic studies, the data are presented as mean ± standard error of the mean (SEM). Statistical significance was calculated by one-way ANOVA, followed by Dunnett’s multiple comparison tests using GraphPad Prism software version 10.2.3 (San Diego, CA, USA). A *p*-value less than 0.05 (*p* < 0.05) was considered statistically significant. Group sizes (ten animals per sex for the main chronic toxicity groups, five per satellite group, five per acute toxicity group, and six per group for the allergic rhinitis experiment) were based on the relevant OECD test guidelines and on previous comparable studies rather than on a formal a priori power calculation. Because the hematological and biochemical screen in the toxicity studies comprised a large number of parameters compared across six groups, the LSD test was used as an exploratory post hoc procedure and no correction for multiple comparisons was applied; the scattered, non-dose-related differences that reached nominal significance, all of which remained within the established reference ranges, are therefore interpreted with caution as probable multiple-testing effects rather than as biologically meaningful, treatment-related changes. Dunnett’s test was used for the in vitro and in vivo anti-allergic analyses because those experiments involved planned comparisons of each treatment group against a single shared control, whereas the broader, exploratory toxicity screen was analyzed with the LSD test.

The experimental unit was the individual animal in the in vivo studies and the individual culture well in the in vitro assay. No predefined inclusion or exclusion criteria were applied, and no animals or data points were excluded from the analyses. Beyond randomization within sex and the coding of cages and treatments, no additional systematic confounders, such as the order of treatments or cage position, were specifically controlled. The studies were conducted under the approved animal ethics protocols (42/2561 and 032/2561); a formal study protocol was not separately pre-registered in a public registry.

## 5. Conclusions

The PCT remedy extract did not cause any symptoms, any signs of toxicity, or death in acute and chronic toxicity tests in rats. The results indicate that the PCT was safe for long-term use. The administration of the ethanolic extract of PCT at concentrations of 75, 150, and 300 mg/kg did not significantly reduce serum OVA-specific IgE, IgG1, or IgG2a, with OVA-specific IgG1 instead being slightly increased at the 300 mg/kg dose. Moreover, all PCT treatments decreased nasal inflammation by reducing the infiltration of inflammatory cells, goblet cells, and mast cells in the nasal epithelium. Additionally, PCT treatments resulted in the downregulation of the Th2 cytokines IL-5 and IL-13 in the nasal mucosa, without a significant change in the Treg cytokine TGF-β. Our findings suggest that PCT has the potential to be considered a therapeutic agent for treating allergic rhinitis (AR). Further research on PCT toxicity should be conducted in non-rodent species, and the biomarkers or active compounds contained in PCT remedies and mechanisms of action and disposition should be isolated and identified.

## Figures and Tables

**Table 1:** Organ weight (g) of female and male rats in chronic toxicity test.

Organ	Control	Satellite Control	PCT Extract			Satellite PCT 1300 mg/kg
			13 mg/kg	130 mg/kg	1300 mg/kg	
**Female rats**						
Brain	2.34 ± 0.08	2.15 ± 0.07	2.34 ± 0.09	2.33 ± 0.06	2.34 ± 0.06	2.03 ± 0.07 *
Lung	2.31 ± 0.16	2.22 ± 0.22	2.08 ± 0.09	2.14 ± 0.09	2.34 ± 0.17	2.46 ± 0.28
Heart	1.23 ± 0.03	1.47 ± 0.12 *	1.20 ± 0.03	1.20 ± 0.04	1.19 ± 0.05	1.43 ± 0.07 *
Liver	13.51 ± 0.34	17.68 ± 1.22 *	13.38 ± 0.28	12.51 ± 0.41	13.87 ± 0.70	16.23 ± 0.98 *
Spleen	0.72 ± 0.03	0.74 ± 0.10	0.70 ± 0.02	0.68 ± 0.03	0.70 ± 0.04	0.71 ± 0.06
Adrenal gland	0.05 ± 0.00	0.04 ± 0.00	0.05 ± 0.02	0.04 ± 0.00	0.04 ± 0.00	0.04 ± 0.00
Kidney	1.53 ± 0.04	1.40 ± 0.08	1.46 ± 0.03	1.56 ± 0.05	1.55 ± 0.05	1.35 ± 0.08 *
Ovary	0.09 ± 0.00	0.08 ± 0.00	0.08 ± 0.00	0.12 ± 0.04	0.10 ± 0.01	0.08 ± 0.01
Uterus	1.11 ± 0.11	1.40 ± 0.13	1.56 ± 0.29	1.36 ± 0.25	0.89 ± 0.07	1.04 ± 0.13
**Male rats**						
Brain	2.38 ± 0.07	2.32 ± 0.07	2.35 ± 0.04	2.36 ± 0.05	2.33 ± 0.07	2.40 ± 0.04
Lung	2.79 ± 0.09	2.03 ± 0.11 *	2.84 ± 0.16	2.96 ± 0.19	2.89 ± 0.17	2.02 ± 0.06 *
Heart	1.66 ± 0.06	1.56 ± 0.06	1.76 ± 0.07	1.77 ± 0.06	1.76 ± 0.09	1.56 ± 0.04
Liver	21.74 ± 0.91	17.10 ± 0.76 *	21.40 ± 1.08	21.97 ± 1.91	23.27 ± 1.08	15.94 ± 0.48 *
Spleen	1.02 ± 0.04	0.99 ± 0.05	1.04 ± 0.06	0.90 ± 0.04	1.03 ± 0.05	0.85 ± 0.02 *
Adrenal gland	0.04 ± 0.00	0.03 ± 0.00	0.04 ± 0.02	0.04 ± 0.00	0.04 ± 0.00	0.03 ± 0.00
Kidney	2.02 ± 0.05	2.03 ± 0.06	2.03 ± 0.06	2.04 ± 0.07	2.10 ± 0.06	1.93 ± 0.06
Testis	1.95 ± 0.02	1.86 ± 0.03	1.69 ± 0.10 *	1.85 ± 0.05	1.79 ± 0.10	1.58 ± 0.16 *
Epididymis	0.93 ± 0.02	0.89 ± 0.03	0.91 ± 0.04	0.97 ± 0.03	0.89 ± 0.03	0.87 ± 0.03

The results are expressed as mean ± standard error of the mean (SEM), *n* = 10. * Significant difference (*p* < 0.05) when compared with control. A satellite group of rats was given distilled water or PCT at 1300 mg/kg/day for 270 days and was then observed for 28 days before being sacrificed, *n* = 5.

**Table 2:** Hematological parameters of female and male rats in chronic toxicity test.

Hematological Parameter	Control	Satellite Control	PCT Extract			Satellite PCT 1300 mg/kg
			13 mg/kg	130 mg/kg	1300 mg/kg	
**Female rats**						
RBC (×10^6^/µL)	7.16 ± 0.13	7.46 ± 0.06	6.96 ± 0.13	7.31 ± 0.12	7.29 ± 0.08	7.56 ± 0.20 *
HB (g/dL)	13.10 ± 0.20	13.58 ± 0.17	13.00 ± 0.23	13.42 ± 0.23	13.62 ± 0.13	14.10 ± 0.22 *
HCT (%)	38.72 ± 1.37	41.86 ± 0.52	39.78 ± 0.81	41.51 ± 0.73	43.89 ± 1.61 *	43.20 ± 0.72 *
MCV (fL)	56.59 ± 0.43	56.14 ± 0.49	57.21 ± 0.52	56.80 ± 0.45	57.98 ± 0.34 *	56.14 ± 0.49
MCH (pg)	18.33 ± 0.14	18.22 ± 0.16	18.70 ± 0.17	18.36 ± 0.13	18.68 ± 0.20	18.68 ± 0.34
MCHC (g/dL)	32.40 ± 0.22	32.64 ± 0.11	33.04 ± 0.44	32.34 ± 0.14	32.22 ± 0.33	32.64 ± 0.11
PLT (×10^5^/µL)	8.14 ± 0.19	8.00 ± 0.30	8.05 ± 0.22	7.46 ± 0.22 *	7.80 ± 0.31	7.86 ± 0.25
WBC (×10^3^/µL)	4.90 ± 0.39	4.93 ± 0.28	4.20 ± 0.37	3.90 ± 0.36	4.94 ± 0.43	3.73 ± 0.34
NEU (×10^3^/µL)	1.54 ± 0.05	1.70 ± 0.07	1.54 ± 0.11	1.51 ± 0.14	2.07 ± 0.34 *	1.23 ± 0.06
LYMP (×10^3^/µL)	2.77 ± 0.31	2.73 ± 0.20	2.28 ± 0.26	2.00 ± 0.20 *	2.36 ± 0.16	2.15 ± 0.31
MONO (×10^3^/µL)	0.44 ± 0.09	0.40 ± 0.10	0.27 ± 0.02 *	0.26 ± 0.04 *	0.36 ± 0.05	0.27 ± 0.03
EOS (×10^3^/µL)	0.15 ± 0.01	0.11 ± 0.01	0.10 ± 0.02	0.13 ± 0.03	0.15 ± 0.03	0.08 ± 0.01
BASO (×10^3^/µL)	0.00 ± 0.00	0.00 ± 0.00	0.00 ± 0.00	0.00 ± 0.00	0.00 ± 0.00	0.00 ± 0.00
**Male rats**						
RBC (×10^6^/µL)	7.83 ± 0.08	7.72 ± 0.10	7.86 ± 0.13	8.06 ± 0.13	7.95 ± 0.10	7.94 ± 0.07
HB (g/dL)	14.16 ± 0.15	14.06 ± 0.18	14.36 ± 0.15	14.54 ± 0.15	14.22 ± 0.15	14.36 ± 0.09
HCT (%)	43.91 ± 0.41	43.34 ± 0.41	43.34 ± 0.41	45.27 ± 0.50 *	44.61 ± 0.50	44.58 ± 0.42
MCV (fL)	56.16 ± 0.28	56.16 ± 0.48	56.16 ± 0.48	56.25 ± 0.59	56.20 ± 0.81	56.18 ± 0.37
MCH (pg)	18.09 ± 0.10	18.22 ± 0.24	18.22 ± 0.24	18.08 ± 0.15	17.90 ± 0.20	18.10 ± 0.11
MCHC (g/dL)	32.23 ± 0.11	32.42 ± 0.22	32.42 ± 0.22	32.11 ± 0.15	31.88 ± 0.16	32.22 ± 0.17
PLT (×10^5^/µL)	8.07 ± 0.41	8.17 ± 0.59	8.17 ± 0.59	7.95 ± 0.25	7.51 ± 0.23	8.10 ± 0.15
WBC (×10^3^/µL)	5.88 ± 0.39	4.94 ± 0.41	5.16 ± 0.20	6.22 ± 0.33	5.48 ± 0.45	4.59 ± 0.45 *
NEU (×10^3^/µL)	1.57 ± 0.14	1.58 ± 0.12	1.57 ± 0.07	1.82 ± 0.12	1.67 ± 0.14	1.49 ± 0.11
LYMP (×10^3^/µL)	3.75 ± 0.37	2.97 ± 0.32	3.15 ± 0.16	3.80 ± 0.23	3.39 ± 0.36	2.80 ± 0.37
MONO (×10^3^/µL)	0.42 ± 0.07	0.25 ± 0.04	0.32 ± 0.05	0.43 ± 0.13	0.29 ± 0.04	0.22 ± 0.01
EOS (×10^3^/µL)	0.15 ± 0.01	0.14 ± 0.01	0.12 ± 0.02	0.16 ± 0.02	0.13 ± 0.02	0.09 ± 0.01 *
BASO (×10^3^/µL)	0.00 ± 0.00	0.00 ± 0.00	0.00 ± 0.00	0.00 ± 0.00	0.00 ± 0.00	0.00 ± 0.00

The results are expressed as mean ± standard error of the mean (SEM), *n* = 10. * Significant difference (*p* < 0.05) when compared with control. A satellite group of rats was given distilled water or PCT at 1300 mg/kg/day for 270 days and was then observed for 28 days before being sacrificed, *n* = 5.

**Table 3:** Biochemical parameters of female and male rats in chronic toxicity test.

Biochemical Parameter	Control	Satellite Control	PCT Extract			Satellite PCT 1300 mg/kg
			13 mg/kg	130 mg/kg	1300 mg/kg	
**Female rats**						
GLU (mg/dL)	159.20 ± 6.33	139.40 ± 7.12	163.60 ± 3.72	148.80 ± 5.06	159.80 ± 6.80	162.40 ± 9.71
BUN (mg/dL)	16.730 ± 1.48	17.28 ± 1.81	16.48 ± 1.44	16.25 ± 1.10	15.50 ± 1.32	18.22 ± 1.08
CRE (mg/dL)	0.84 ± 0.03	0.86 ± 0.05	0.84 ± 0.02	0.88 ± 0.04	0.84 ± 0.02	0.89 ± 0.04
TP (g/dL)	7.14 ± 0.14	7.12 ± 0.17	7.41 ± 0.08	7.38 ± 0.14	7.11 ± 0.11	7.68 ± 0.19 *
ALB (g/dL)	3.73 ± 0.07	3.80 ± 0.08	3.88 ± 0.04	3.91 ± 0.06	3.67 ± 0.09	4.06 ± 0.08
T-BIL (mg/dL)	0.13 ± 0.01	0.10 ± 0.03	0.31 ± 0.01	0.16 ± 0.02	0.14 ± 0.03	0.13 ± 0.02
D-BIL (mg/dL)	0.06 ± 0.01	0.05 ± 0.00	0.08 ± 0.00	0.08 ± 0.00	0.07 ± 0.00	0.08 ± 0.00
AST (U/L)	80.70 ± 5.03	89.40 ± 14.78	83.20 ± 10.59	86.90 ± 3.88	86.90 ± 3.88	97.60 ± 14.40
ALT (U/L)	32.10 ± 4.30	34.20 ± 8.59	32.30 ± 5.19	31.70 ± 1.88	31.70 ± 1.88	35.80 ± 13.06
ALP (U/L)	29.10 ± 1.64	22.60 ± 1.72	25.60 ± 1.86	34.90 ± 4.29	34.90 ± 4.29	22.80 ± 3.25
**Male rats**						
GLU (mg/dL)	183.20 ± 4.61	168.80 ± 7.13	166.70 ± 3.94 *	169.70 ± 5.18 *	170.80 ± 4.24	166.00 ± 5.13 *
BUN (mg/dL)	9.83 ± 0.49	9.84 ± 0.62	9.86 ± 0.38	10.87 ± 0.36	11.22 ± 0.69	9.48 ± 0.22
CRE (mg/dL)	0.69 ± 0.02	0.70 ± 0.03	0.73 ± 0.01	0.70 ± 0.02	0.72 ± 0.03	0.77 ± 0.01
TP (g/dL)	5.85 ± 0.07	6.40 ± 0.09	5.68 ± 0.08	5.91 ± 0.07	5.76 ± 0.08	6.20 ± 0.03 *
ALB (g/dL)	2.96 ± 0.02	3.02 ± 0.02	2.91 ± 0.03	2.99 ± 0.02	2.93 ± 0.04	3.02 ± 0.02
T-BIL (mg/dL)	0.07 ± 0.00	0.13 ± 0.00 *	0.08 ± 0.01	0.07 ± 0.01	0.07 ± 0.00	0.15 ± 0.01 *
D-BIL (mg/dL)	0.06± 0.00	0.08 ± 0.00 *	0.05 ± 0.00	0.06 ± 0.00	0.05 ± 0.00	0.07 ± 0.00 *
AST (U/L)	88.70 ± 7.46	102.60 ± 13.53	82.80 ± 4.58	95.40 ± 5.71	104.10 ± 12.84	102.20 ± 5.92
ALT (U/L)	32.00 ± 3.16	34.60 ± 5.07	29.00 ± 1.20	37.60 ± 5.41	45.90 ± 10.29	30.20 ± 3.51
ALP (U/L)	58.00 ± 2.72	59.40 ± 7.29	55.00 ± 2.19	57.90 ± 5.62	66.80 ± 8.24	57.20 ± 3.28

The results are expressed as mean ± standard error of the mean (SEM), *n* = 10. * Significant difference (*p* < 0.05) when compared with control. A satellite group of rats was given distilled water or PCT at 1300 mg/kg/day for 270 days and was then observed for 28 days before being sacrificed, *n* = 5.

## Data Availability

The data presented in this study are available on request from the corresponding author.

## Supplementary Materials

The following supporting information can be downloaded at: https://www.mdpi.com/article/10.3390/ijms27157031/s1.

## Abbreviations

The following abbreviations are used in this manuscript:

PCT	Prabchompoothaweep
TTM	Thai traditional medicine
OVA	Ovalbumin
Th2	T helper 2
AR	Allergic rhinitis
NLEM	National List of Essential Medicines
CPM	Chlorpheniramine
SEM	Standard error of the mean
H&E	Hematoxylin and Eosin
PAS	Periodic Acid-Schiff
TB	Toluidine blue
Th1	T helper 1
Treg	Regulatory T
IL	Interleukin
IFN	Interferon
TGF	Transforming growth factor
IC_50_	Inhibitory concentration of 50%
