# Supplementary material for: Acute and Chronic Toxicity Tests and Assessment of In Vitro and In Vivo Anti-Allergic Effects of Prabchompoothaweep Remedy
Source: Int J Mol Sci. 2026 Aug 5;27(15):7031. doi: 10.3390/ijms27157031 (PMC13466349; doi:10.3390/ijms27157031)
Supplement: Supplementary file 1 [file ijms-27-07031-s001.zip › ijms-4378349-supplementary.pdf]

Supplementary Materials

Contents:

Table S1. The plant ingredients, voucher specimen number and proportion of Prabchompoothaweep remedy.

Table S2. The percentage yield and inhibition on β-hexosaminidase release of the PCT ethanolic extract and its plant ingredients from RBL-2H3 cells (n = 3).

Table S3. Body weight (g) of female rats in acute toxicity test.

Table S4. Organ weight (g) of female rats in acute toxicity test.

Table S5. Grades of histological descriptions in mouse nasal tissues after staining with (A) Hematoxylin and Eosin (H & E) dyes and (B) Periodic Acid-Schiff (PAS) dye.

Table S6. Oligonucleotide primers used for quantitative real-time PCR of monitoring cytokine gene expressions.

Figure S1. Piperine content in PCT remedy by using HPLC (A) The peak of standard Piperine at concentration of 100 µg/mL, (B) The fingerprint of 95% ethanolic extract of PCT, and (C) The standard Piperine mixed with PCT at concentration of 100 µg/mL.

Table S1. The plant ingredients, voucher specimen number and proportion of Prabchompoothaweep remedy.

Scientific name	Family	Thai name	Voucher specimen number	Part used	Proportion (%w/w)
Acanthus ebracteatus Vahl.	Acanthaceae	Ngueak pla mo	SKP 001 01 05 01	Aerial part	25.81
Amomum testaceum Ridl.	Zingiberaceae	Krawan	SKP 206 01 11 01	Fruit	0.21
Amorphophallus saraburiensis (Dennst.)	Araceae	Buk ro	SKP 015 01 19 01	Rhizome	1.72
Anethum graveolens L.	Umbelliferae	Thian ta takkataen	SKP 199 01 07 01	Seed	1.29
Angelica dahurica (Hoffm.) Benth. & Hook.f. ex Franch. & Sav. var. *dahurica*	Umbelliferae	Kot so	SKP 199 01 19 01	Root	0.86
Ardisia polycephala Wall. ex A.DC.	Myrsinaceae	Philangkasa	SKP 122 01 16 01	Fruit	0.86
Atractylodes lancea (Thunb.) DC.	Compositae	Kot kamao	SKP 051 01 12 01	Rhizome	0.86
Cinnamomum camphora (L.) J.S. Presl	Lauraceae	Karabun	-	Camphor	0.43
Clausena excavata Burm. f.	Rutaceae	Hasakhun thet	SKP 183 11 08 01	Stem	2.15
Enhalus acoroides (L.f) Rovle.	Hydrocharitaceae	Lamphan hangmu	SKP 088 05 01 01	Rhizome	0.86
Foeniculum vulgare Mill.	Umbelliferae	Thian klaep	SKP 199 06 22 01	Seed	1.29
Leonurus sibiricus L.	Lamiaceae	Kancha thet	SKP 095 12 19 01	Aerial part	25.81
Lepidium sativum L.	Cruciferae	Thian daeng	SKP 019 12 19 01	Seed	1.29
Myristica fragrans Houtt.	Myristicaceae	Look chan or Nutmeg	SKP 121 13 06 01	Seed	0.21
Myristica fragrans Houtt.	Myristicaceae	Dok chan or Mace	SKP 121 13 06 01	Aril	0.21
Nigella sativa L.	Ranunculaceae	Thian dam	SKP 160 14 19 01	Seed	0.86
Piper nigrum L.	Piperaceae	Phrikthai dam	SKP 146 16 14 01	Seed	25.81
Piper retrofractum Vahl.	Piperaceae	Dee plee	SKP 146 16 18 01	Flower	0.43
Plumbago indica L.	Plumbaginaceae	Chetta mun phloeng daeng	SKP 148 16 09 01	Root	1.72
Syzygium aromaticum (L.) Merr. & L.M. Perry	Myrtaceae	Kan phlu	SKP 123 19 01 01	Flower	2.15
Terminalia arjuna Wight and Arn.	Combretaceae	Samo thet	SKP 049 20 01 01	Fruit	1.72
Terminalia chebula Retz.	Combretaceae	Samo thai	SKP 049 20 03 01	Fruit	1.72
Zingiber officinale Roscoe	Zingiberaceae	Khing	SKP 206 26 15 01	Rhizome	1.72

Table S2. The percentage yield and inhibition on β-hexosaminidase release of the PCT ethanolic extract and its plant ingredients from RBL-2H3 cells (n = 3).

Scientific name	Code	%Yield (w/w)	Inhibition of β-hexosaminidase release (%) at various concentrations (μg/mL)				IC_{50} ± SEM (μg/mL)
			1	10	50	100	
A. ebracteatus	AEE	4.97	12.80 ± 6.75	34.98 ± 3.94	58.09 ± 3.20	78.66 ± 0.14	19.49 ± 1.76
A. testaceum	ATE	1.52	10.24 ± 0.11	29.30 ± 1.40	45.61 ± 0.86	66.12 ± 0.84	69.33 ± 2.63*
A. saraburiensis	ASE	1.62	0.28 ± 7.41	21.70 ± 5.21	44.72 ± 1.94	71.40 ± 1.74	66.52 ± 3.09*
A. graveolens	AGE	5.70	-3.24 ± 3.02	21.45 ± 1.70	38.50 ± 1.78	57.70 ± 1.97	88.46 ± 1.80*
A. dahurica	ADE	2.66	4.30 ± 2.84	34.01 ± 1.23	64.24 ± 0.32	83.76 ± 3.97	17.08 ± 0.34
A. polycephala	APE	7.02	5.61 ± 2.64	34.83 ± 0.78	60.18 ± 1.14	79.36 ± 2.73	17.17 ± 0.97
A. lancea	ALE	12.67	7.52 ± 7.86	33.58 ± 3.86	64.28 ± 4.96	85.33 ± 6.45	18.15 ± 0.83
C. camphora	CCE	NE	7.48 ± 1.57	23.86 ± 3.02	46.04 ± 1.50	67.19 ± 5.51	64.50 ± 2.77*
C. excavata	CEE	1.44	10.55 ± 6.09	70.07 ± 3.65	88.27 ± 3.08	96.34 ± 0.94	6.82 ± 0.23*
E. acoroides	EAE	2.80	1.94 ± 9.63	23.06 ± 2.38	40.94 ± 4.08	66.50 ± 4.40	74.23 ± 6.44*
F. vulgare	FVE	3.02	1.36 ± 7.13	29.95 ± 2.73	49.00 ± 2.98	61.93 ± 3.711	21.68 ± 1.06
L. sibiricus	LSE(K)	15.74	6.34 ± 7.50	37.48 ± 3.65	69.68 ± 3.13	93.17 ± 1.90	15.35 ± 2.01
L. sativum	LSE(T)	10.12	-8.17 ± 4.80	12.80 ± 2.48	24.84 ± 2.87	57.40 ± 2.02	92.69 ± 1.78*
M. fragrans (Nutmeg)	MFE(N)	5.00	25.36 ± 6.25	45.68 ± 4.55	78.67 ± 2.81	96.41 ± 0.83	12.04 ± 2.41*
M. fragrans (Mace)	MFE(M)	19.15	11.37 ± 4.52	35.02 ± 2.52	61.82 ± 4.88	86.43 ± 7.41	18.86 ± 1.32

N. sativa	NSE	18.93	-0.64 ± 1.38	11.54 ± 2.00	30.51 ± 2.40	43.05 ± 0.98	> 100*
P. nigrum	PNE	5.62	9.90 ± 0.80	44.25 ± 4.26	83.62 ± 4.97	101.90 ± 1.56	12.30 ± 1.85*
P. retrofractum	PRE	14.05	24.73 ± 2.65	52.20 ± 6.63	80.60 ± 10.30	97.81 ± 3.77	9.97 ± 2.15*
P. indica	PIE	11.42	11.39 ± 4.13	47.74 ± 2.16	94.42 ± 3.41	102.5 ± 0.96	10.88 ± 0.66*
S. aromaticum	SAE	35.88	-1.87 ± 4.68	31.09 ± 0.42	50.17 ± 2.40	79.24 ± 4.28	19.31 ± 1.57
T. arjuna	TAE	41.10	-0.34 ± 3.71	25.46 ± 1.79	61.80 ± 1.74	80.34 ± 1.83	24.26 ± 0.55
T. chebula	TCE	46.38	33.88 ± 1.25	47.78 ± 0.92	69.88 ± 1.18	87.96 ± 1.74	12.37 ± 1.25*
Z. officinale	ZOE	12.76	3.69 ± 3.42	38.06 ± 5.45	69.55 ± 0.66	90.46 ± 2.84	14.84 ± 2.79
Prabchompoothaweep	PCT	12.80	16.60 ± 8.06	43.24 ± 5.02	65.20 ± 1.16	78.23 ± 4.43	13.18 ± 2.67*
Chlorpheniramine	CPM	NE	1.98 ± 2.55	26.61 ± 2.24	72.80 ± 0.93	95.14 ± 1.20	22.12 ± 1.07

Note: NE means not extracted. Data were analyzed by one-way ANOVA and Dunnett's multiple comparison tests. * Significant difference ($p < 0.05$) when compared with chlorpheniramine (a positive control drug).

Table S3. Body weight (g) of female rats in acute toxicity test.

Group	Body weight (g)		
	**Day 1**	**Day 7**	**Day 14**
Control	202.00 ± 2.55	214.00 ± 5.34	221.00 ± 4.85
PCT 5,000 mg/kg	201.00 ± 1.87	194.00 ± 4.30*	193.00 ± 6.63*

The results are expressed as mean ± standard error of the mean (SEM), n = 5. * Significant difference ($p < 0.05$) when compared with control.

Table S4. Organ weight (g) of female rats in acute toxicity test.

Organ	Organ weight (g)	
	**Control**	**PCT 5,000 mg/kg**
Brain	1.80 ± 0.02	1.76 ± 0.02
Lung	1.10 ± 0.01	0.96 ± 0.04*
Heart	0.82 ± 0.02	0.68 ± 0.02*
Liver	7.83 ± 0.21	6.53 ± 0.19*
Spleen	0.86 ± 0.02	0.64 ± 0.04*
Adrenal gland	0.03 ± 0.00	0.03 ± 0.00
Kidney	0.86 ± 0.02	0.68 ± 0.03*
Ovary	0.06 ± 0.00	0.05 ± 0.00
Uterus	0.58 ± 0.06	0.33 ± 0.03*

The results are expressed as mean ± standard error of the mean (SEM), n = 5. * Significant difference ($p < 0.05$) when compared with control.

Table S5. Grades of histological descriptions in mouse nasal tissues after staining with (A) Hematoxylin and Eosin (H & E) dyes and (B) Periodic Acid-Schiff (PAS) dye.

**(A) H & E staining**	
**Grade**	**Histological descriptions**
1	Epithelial cells normal with no or some inflammatory cells infiltration into nasal tissue (not more than 10)
2	Some epithelial cells degenerate, moderate inflammatory cells infiltration into nasal tissue (between 10 and 20)
3	Some epithelial cells necrosis or detached from basal line, more inflammatory cells infiltrated into nasal tissue than grade 2 (more than 20)
4	Almost of epithelial cells necrosis with a lot of inflammatory cells accumulate in nasal cavity
**(B) PAS staining**	

Grade	Histological descriptions
1	Light magenta color of mucus glands in nasal tissues (+)
2	Magenta color of mucus glands in nasal tissues (++)
3	Dark magenta color of mucus glands in nasal tissues (+++)

Table S6. Oligonucleotide primers used for quantitative real-time PCR of monitoring cytokine gene expressions.

Gene	Primer	Size of PCR product (bp)
IL-5	F: 5'-ATGATCGTGCCTCTGTGCCTGGAGC-3'	242
	R: 5'-CTGTTTTTCCTGGAGTAAACTGGGG-3'	
IL-13	F: 5'-AGACCAGACTCCCCTGTGCA-3'	123
	R: 5'-TGGGTCCTGTAGATGGCATTG-3'	
IL-12b (p40)	F: 5'-GGAAGCACGGCAGCAGAATA-3'	180
	R: 5'-AACTTGAGGGAGAAGTAGGAATGG-3'	
TGF-β	F: 5'-CAAGGGCTACCATGCCAACT-3'	84
	R: 5'-AGGGCCAGGACCTTGCTG-3'	
β-actin	F: 5'-GGCCAACCGTGAAAAGATGA-3'	251
	R: 5'-CACGCTCGGTCAGGATCTTC-3'	

(A)

(B)

(C)

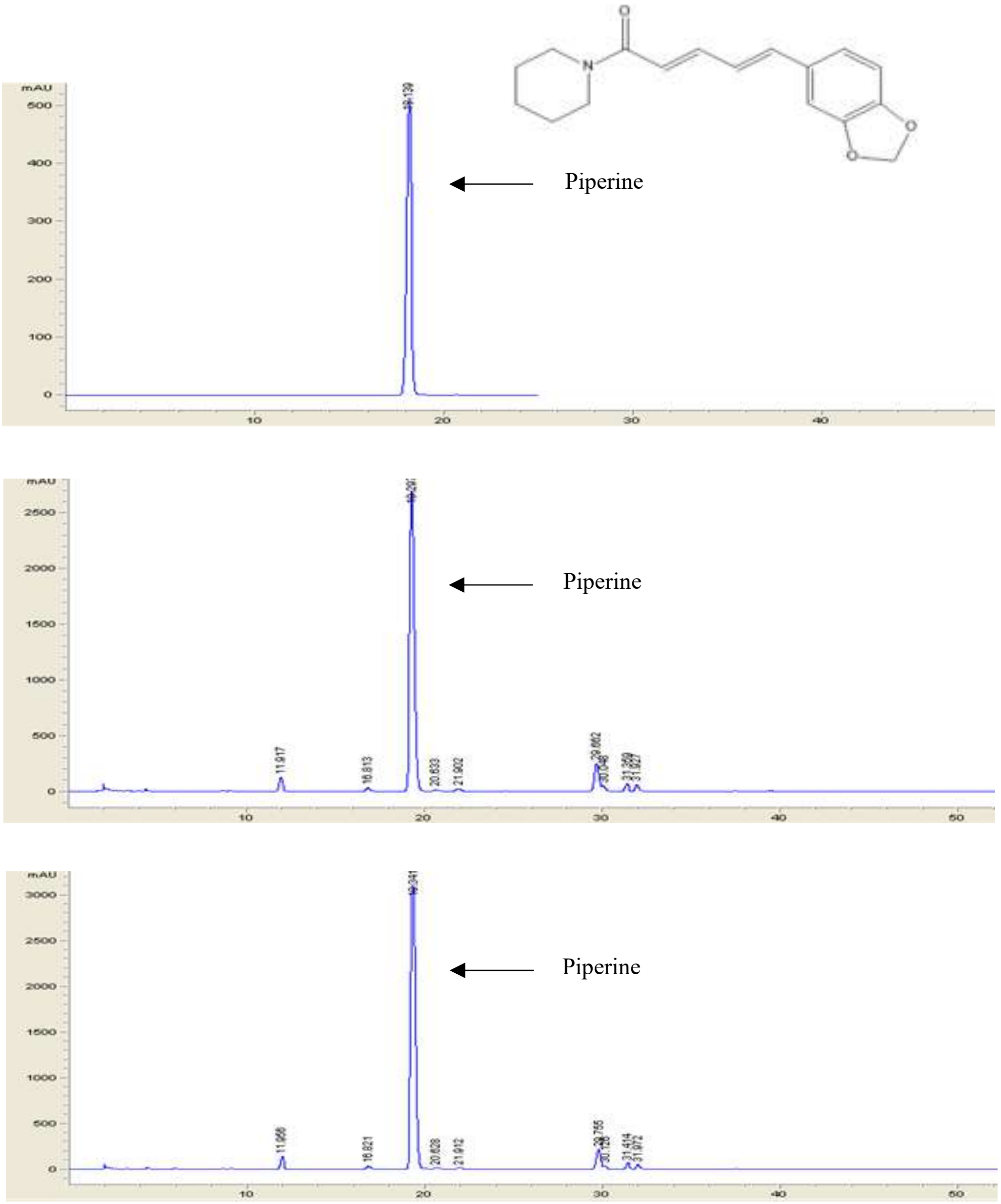

Figure S1. Piperine content in PCT remedy by using HPLC (A) The peak of standard Piperine at concentration of 100 µg/mL, (B) The fingerprint of 95% ethanolic extract of PCT, and (C) The standard Piperine mixed with PCT at concentration of 100 µg/mL.
